# The Oxford Positive Self Scale: psychometric development of an assessment of cognitions associated with psychological well-being

**DOI:** 10.1017/S0033291723000624

**Published:** 2023-11

**Authors:** Daniel Freeman, Laina Rosebrock, Bao S. Loe, Simone Saidel, Jason Freeman, Felicity Waite

**Affiliations:** 1 Department of Experimental Psychology, University of Oxford, Oxford, UK; 2 Oxford Health NHS Foundation Trust, Oxford, UK; 3 The Psychometrics Centre, University of Cambridge, Cambridge, UK

**Keywords:** Cognitions, psychological well-being, psychometrics, questionnaire

## Abstract

**Background:**

Developing, elaborating, and consolidating positive views of the self is a plausible route to increased psychological well-being. We set out to provide an assessment of positive self-beliefs that could be used in research and clinical practice.

**Methods:**

A non-probability online survey was conducted with 2500 UK adults, quota sampled to match the population for age, gender, ethnicity, income, and region. Exploratory factor analysis of a 94-item pool – generated with guidance from people with lived experience of mental health difficulties – was conducted to develop the Oxford Positive Self Scale (OxPos). The item pool was further reduced using regularised structural equation modelling (SEM) before confirmatory factor analysis. Optimal cut-off scores were developed using receiver operating characteristic curves. Additional validations were carried out with two further general population cohorts (*n* = 1399; *n* = 1693).

**Results:**

A 24-item scale was developed with an excellent model fit [robust χ^2^ = 995.676; df = 246; CFI = 0.956; TLI = 0.951; RMSEA = 0.049 (0.047, 0.052); SRMR = 0.031]. The scale comprises four factors: mastery; strength; enjoyment; and character. SEM indicated that the scale explains 68.6% of variance in psychological well-being. The OxPos score was negatively correlated with depression (*r* = −0.49), anxious avoidance (*r* = −0.34), paranoia (*r* = −0.23), hallucinations (*r* = −0.20), and negative self-beliefs (*r* = −0.50), and positively correlated with psychological well-being (*r* = 0.79), self-esteem (*r* = 0.67), and positive social comparison (*r* = 0.72). Internal reliability and test–retest reliability were excellent. Cut-offs by age and gender were generated. A short-form was developed, explaining 96% of the full-scale variance.

**Conclusions:**

The new open access scale provides a psychometrically robust assessment of positive cognitions that are strongly connected to psychological well-being.

## Introduction

In determining psychological health, views of the self are central. Negative and positive views of the self, although inversely correlated, are distinct constructs that studies have shown are separate factors (Bryant & Baxter, 1997; Faustino, 2022; Fowler et al., 2006). Negative views of the self are an important element in understanding and treating mental health conditions. For example, cognitions concerning the self as a failure or unlikeable are considered a central cause of depression (Beck, 1963). Such cognitions are then targeted in psychological interventions (Kovacs, Rush, Beck, & Hollon, 1981). Positive views of the self have less often been a focus of research. They have mainly been viewed – often within the field of positive psychology – as a factor in developing psychological well-being (Seligman, 2019). We similarly view positive self-beliefs as a route to psychological well-being, but also believe their development in mental health treatment can function as a potential counter-weight to negative self-perceptions. That is, they offer an alternative way to address low self-esteem. Accurate measurement of the factor of interest is essential for successful research and clinical practice. In this paper we report the development in a representative general population sample of a measure of positive self-beliefs that highlights potentially tractable cognitions to improve psychological well-being.

The dual continua model hypothesis proposes that mental health (psychological well-being) and mental ill health (disorders) are two separate but correlated dimensions (Keyes, 2013). Twin study data support this view of shared but also separate genetic and environmental causation for internalising disorders and psychological well-being (Kendler, Myers, Maes, & Keyes, 2011). A similar perspective can be taken for negative and positive views of the self. Negative self-beliefs are likely to mean fewer positive self-beliefs. Nevertheless, the presence of negative self-beliefs does not imply the absence of positive self-beliefs. Similarly, the absence of negative self-beliefs does not guarantee the presence of positive self-beliefs. There are many scales across mental health research that assess negative thoughts, for example the Automatic Thoughts Questionnaire (Hollon & Kendall, 1980) and the Cognition Checklist (Beck, Brown, Steer, Eidelson, & Riskind, 1987). Many other scales include negative thoughts within disorder-specific understandings, such as in the Posttraumatic Cognitions Inventory (Foa, Ehlers, Clark, Tolin, & Orsillo, 1999). Despite a focus on negative cognitions, the importance of positive cognitions has always been recognised, and counter-part scales such as the Positive Automatic Thoughts Questionnaire (ATQ-P) (Ingram, Kendall, Siegle, Guarino, & McLaughlin, 1995; Ingram & Wisnicki, 1988) have been developed. The ATQ-P comprises 30-items forming four factors described as positive daily functioning (e.g. ‘Life is exciting’), positive self-evaluation (e.g. ‘I take good care of myself’), others evaluation of self (e.g. ‘I have a good sense of humour’), and positive future expectations (e.g. ‘My future looks bright’). The Brief Core Schema Scale (Fowler et al., 2006) includes assessment of six positive self beliefs (e.g. ‘I am respected’ ‘I am valuable’ ‘I am talented’) that form a single factor. The Questionnaire about the Process of Recovery (Law, Neil, Dunn, & Morrison, 2014) comprises 15-items (e.g. ‘I feel better about myself’, ‘I feel able to take chances in life’ ‘I am able to develop positive relationships with other people’), developed from interviews about recovery from psychosis, that form a single factor.

However, in our view interventions designed to promote positive self-beliefs in order to enhance psychological well-being require –and currently lack – a scale that can select those in need, guide development of the intervention, and monitor outcomes. Such a scale would need to: be informed by people with lived experience of mental health problems; assess the cognitions highly connected to psychological well-being; assess the types of positive self-cognitions that are tractable with psychological approaches; be developed in a large representative cohort of the general population; enable interpretation by age and gender; be readily useable; and offer excellent reliability and validity. We set out to develop such a scale.

## Methods

### Participants

An online survey with a quota sampled UK participant group of 2500 adults (16 + years old) was conducted from 21^st^ June 2022 to 28^th^ July 2022 via a market research company. The quotas were based on UK Office for National Statistics population estimate data for gender, age, ethnicity, income, and region. Invited respondents did not know the topic of the survey before provisional agreement to complete it. Data from a further 1399 individuals who completed the survey via the market research company but did not form part of the representative cohort became an additional validation sample, as did a further 1693 individuals who were recruited separately via social media advertisements (the test-re-test group was drawn from this latter cohort). Ethical approval was obtained from the University of Oxford Medical Sciences Interdivisional Research Ethics Committee. Written informed consent was obtained from all participants.

### Assessments

#### The Oxford Positive Self Scale (OxPos)

A pool of 94 items (see online Supplementary materials) assessing positive cognitions about the self was developed by the research group and a Lived Experience Advisory Panel (LEAP) of 14 young people with lived experience of psychosis, and there was also consideration of existing scales, particularly the Brief Core Schema Scale (Fowler et al., 2006) and Social Comparison Scale (Allan & Gilbert, 1995). Items were generated in the context of developing a new automated VR therapy (Phoenix) to enhance positive cognitions particularly for patients diagnosed with psychosis. Therefore there was a focus on positive cognitions that could be plausible targets in psychological intervention. Each scale item was rated on a scale comprising: Do not believe it (0), Believe it slightly (1), Believe it moderately (2), Believe it very much (3), and Believe it totally (4). Participants were asked to try to judge the beliefs on how they viewed themselves over the past week. Higher scores indicate greater endorsement of items. The final scale and the short-form are provided in online Supplementary materials.

#### Warwick-Edinburgh Mental Well-being Scale (WEMWBS)

(Tennant et al., 2007). The WEMWBS is a fourteen-item scale assessing psychological well-being over the past fortnight. Each item is rated on a 1 (none of the time) to 5 (all of the time) scale, and therefore the total score can range from 14 to 70, with higher scores indicating a greater level of well-being. The Cronbach's alpha was 0.95.

#### Patient Health Questionnaire (PHQ-9)

(Kroenke, Spitzer, & Williams, 2001). This scale assesses depressive symptoms over the past fortnight. Each of the nine items is rated on a 0 (not at all) to 3 (nearly every day) scale. Higher scores indicate higher levels of depression. The Cronbach's alpha was 0.92.

#### Oxford agoraphobic avoidance scale (O-AS)

(Lambe et al., 2021). The O-AS lists eight simple tasks progressing in difficulty. Participants are asked whether they could do the task now or whether they could not because of anxiety (Yes = 0, No = 1), which provides the avoidance score (0–8). For each task participants are also asked on a 0 (no distress) to 10 (extreme distress) scale how anxious they would feel doing it. These distress scores are summed to provide an overall distress score. Higher scores indicate greater agoraphobic symptoms. The Cronbach's alpha was 0.91.

#### Revised green *et al*. paranoid thoughts scale (R-GPTS)

(Freeman et al., 2021). The R-GPTS comprises an eight-item ideas of reference scale and a 10-item ideas of persecution scale. Each item is rated for the past two weeks on a 5-point (0 to 4) scale. Higher scores indicate greater levels of paranoia. The Cronbach's alpha was 0.97.

#### Cardiff anomalous perceptions scale-hallucinations (CAPS)

(Bell, Halligan, & Ellis, 2006). This scale comprises eleven hallucination items taken from the CAPS. Each item (e.g. ‘Hear voices commenting on what you're thinking or doing’) is rated on a 0 (not at all) to 5 (daily) scale. Higher scores indicate greater levels of hallucinatory experiences. The Cronbach's alpha was 0.96.

#### Brief core schema scales (BCSS)

(Fowler et al., 2006). The six-item negative (e.g. ‘I am unloved’, ‘I am worthless’) and the six-item positive self-belief (e.g. ‘I am successful’ ‘I am good’) sub-scales were used. Each item is rated on a scale from 0 (do not believe it) to 4 (believe it totally). Higher scores indicate greater endorsement of items. The Cronbach's alpha was 0.91.

#### Social comparison scale (SCS)

(Allan & Gilbert, 1995). This measure comprises nineteen bipolar scales (e.g. inferior–superior, incompetent–competent, unlikeable–likeable) asking people to rate how they feel in comparison to others. Each was rated on a six-point Likert scale from ‘highly [e.g. inferior]’ to ‘highly [e.g. superior]’. Higher scores indicate a more positive view of the self in relation to others. The Cronbach's alpha was 0.97.

#### Rosenberg self-esteem scale (RSES)

(Rosenberg, 1965). The RSES is a 10-item, four-point scale (1–4) that assesses current levels of global self-esteem. The scale was scored so that a high total score was indicative of higher global self-esteem. The Cronbach's alpha was 0.89.

### Analysis

Analyses were conducted in R, version 4.2.1 (R Core Team, 2022) with the psych (Revelle, 2020), lavaan (Rosseel, 2012), semTools (Jorgensen et al., 2016), regSEM (Jacobucci, 2017) and cutpointr (Thiele & Hirschfeld, 2021) packages. We divided the data randomly and equally into two groups: an evaluation sample (*n* = 1250) and a validation sample (*n* = 1250). Exploratory factor analysis (EFA) was performed on the evaluation sample, and confirmatory factor analysis (CFA) on the validation sample. Prior to EFA, Bartlett's Test of Sphericity and the Kaiser-Mayer Olkin Measure of Sampling Adequacy (KMO) were used to evaluate the feasibility of factor recovery given the dataset (Bartlett, 1950; Kaiser, 1970). We used parallel analysis to determine the number of factors to retain.

EFA using Pearson correlations with oblique rotation and minimum residual factoring assessed the factorial structure of the new scale. Minimum residual was selected as the factoring method because of its robustness to non-normal data (Fabrigar, Wegener, MacCallum, & Strahan, 1999). Criteria used to retain items were based on factor loadings (>0.5), clarity of item content, and the theoretical coherence of subscales. CFA based on the robust maximum likelihood (MLR) estimator was subsequently carried out. Model goodness of fit was based on recommended guidelines where CFI and TLI >0.90 (Bentler, 1990), RMSEA <0.08 (Brown & Cudeck, 1993), and SRMR <0.08 (Hooper, Coughlan, & Mullen, 2008). The item pool was further reduced using regularised SEM (regSEM) with psychological well-being as the primary outcome (Jacobucci et al., 2016) (see Section 1 of online supplementary materials for full details). The final set of items retained from regSEM analyses was re-evaluated using CFA. (We also performed the final CFA model on the two additional non-representative datasets to validate the model's goodness of fit.) We performed measurement invariance (MI) analyses based on age and gender to evaluate the items for potential bias (see Section 2 of online supplementary materials). We employed a higher-order CFA model to assess the appropriateness of calculating a total score. Internal consistency of the factors was assessed using omega coefficient (*ω*), and a one-week test–retest reliability based on 659 individuals repeating the questionnaire was evaluated by a two-way consistency intraclass correlation coefficient.

We created a short version of the new measure, in which two items were selected from each factor. The items were selected based on the highest factor loadings while ensuring that the item content was not too similar. We assessed the goodness-of-fit of the model based on the correlated factor model and the higher-order CFA model. The internal consistency of the factors was assessed using omega coefficient – *ω* (McDonald, 2013), and the overall score of the short version was correlated with the full version of the scale.

We conducted separate structural equation modelling (SEM) regressions between the new (long and short) scale factors and the well-being factor using the complete dataset. Furthermore, to identify the most important factors that predict well-being, accounting for shared variance, we also performed multiple SEM regressions using both long and short versions of the scale. Convergent and discriminant validity with the other scales was assessed using Pearson correlation coefficients.

Finally, optimal cut-off scores for the Oxford Positive Self Scale (OxPos) were developed using the receiver operating characteristic (ROC) curves (see online supplementary materials for further details). The ROC analyses were conducted using the cut-off points on the WEMWBS. We used the maximisation metric to maximise sensitivity and specificity. Specifically, the optimal cut-off score was calculated based on Youden's J statistic (Youden, 1950), which occurs at the point which is furthest from the ROC curve and is interpreted as the maximum vertical distance between the ROC curve and the chance line (Schisterman, Perkins, Liu, & Bondell, 2005).

## Results

A summary of the socio-demographic characteristics of the participants is provided in Table 1.

**Table 1.** Socio-demographic information

	Representative sample (*n* = 2500)	Non-representative sample (*n* = 1399)	Social media sample (*n* = 1693)
	Mean (s.d.)/*n* (%)	Mean (s.d.)/*n* (%)	Mean (s.d.)/*n* (%)
Age in years	45.4 (16.3)	38.5 (14.8)	49.1 (20.9)
Age ranges			
16–24	323 (12.9%)	249 (17.8%)	395 (23.6%)
25–34	430 (17.2%)	387 (27.7%)	119 (7.0%)
35–44	463 (18.5%)	368 (26.3%)	75 (4.4%)
45–54	407 (16.3%)	176 (12.6%)	200 (11.8%)
55–64	517 (20.7%)	121 (8.6%)	393 (23.2%)
65+	357 (14.3%)	95 (6.8%)	494 (29.2%)
Gender: Male; Female; Other; Prefer not to say	1213; 1274; 12; 1	659; 730; 3; 7	207; 1428; 38; 20
Ethnicity:			
White			
English/Welsh/Scottish/Northern Irish/British	1910 (76.4%)	1025 (73.3%)	1442 (85.2%)
Irish	29 (1.2%)	27 (1.9%)	24 (1.4%)
Gypsy or Irish Traveller	7 (0.3%)	3 (0.2%)	1 (0.1%)
Any other White background	60 (2.4%)	64 (4.6%)	110 (6.5%)
Mixed/Multiple ethnic groups			
White and Black Caribbean	22 (0.8%)	28 (2.0%)	13 (0.8%)
White and Black African	10 (0.4%)	9 (0.6%)	5 (0.3%)
White and Asian	12 (0.5%)	20 (1.4%)	18 (1.1%)
Any other Mixed/Multiple ethnic background	13 (0.5%)	66 (4.7%)	17 (1.0%)
Asian/Asian British			
Indian	96 (3.8%)	59 (4.2%)	21 (1.2%)
Pakistani	109 (4.4%)	64 (4.6%)	15 (0.9%)
Bangladeshi	52 (2.1%)	23 (1.6%)	4 (0.2%)
Chinese	47 (1.9%)	18 (1.3%)	19 (1.1%)
Any other Asian background	43 (1.7%)	33 (2.4%)	14 (0.8%)
Black/African/Caribbean/Black British			
African	107 (4.3%)	91 (6.5%)	8 (0.5%)
Caribbean	52 (2.1%)	27 (1.9%)	5 (0.3%)
Any other Black/African/Caribbean background	7 (0.3%)	12 (0.9%)	2 (0.1%)
Other ethnic group			
Arab	19 (0.8%)	30 (2.1%)	5 (0.3%)
Any other ethnic group	18 (0.7%)	17 (1.2%)	9 (0.5%)
Marital status:			
Single	783 (31.6%)	545 (39.2%)	537 (31.7%)
Cohabiting	247 (10.0%)	198 (14.3%)	168 (9.9%)
Married or civil partnership	1181 (47.6%)	513 (36.9%)	677 (40.0%)
Divorced or separated	195 (7.9%)	85 (6.1%)	181 (10.7%)
Widowed	56 (2.3%)	34 (2.4%)	87 (5.1%)
Prefer not to say	19 (0.8%)	14 (1.0%)	42 (2.5%)
Total household income:			
Less than £ 10 000	201 (8.1%)	198 (14.2%)	209 (12.3%)
£ 10 000–£ 19 999	526 (21.1%)	223 (16.0%)	147 (8.7%)
£ 20 000–£ 29 999	508 (20.4%)	323 (23.1%)	287 (17.0%)
£ 30 000–£ 39 999	378 (15.2%)	247 (17.7%)	209 (12.3%)
£ 40 000–£ 49 999	254 (10.2%)	238 (17.0%)	135 (8.0%)
£ 50 000–£ 59 999	211 (8.5%)	39 (2.8%)	125 (7.4%)
£ 60 000–£ 69 999	111 (4.5%)	29 (2.1%)	90 (5.3%)
£ 70 000–£ 99 999	162 (6.5%)	27 (1.9%)	122 (7.2%)
£ 100 000 and above	76 (3.1%)	52 (3.7%)	98 (5.8%)
Prefer not to say	62 (2.5%)	21 (1.5%)	268 (15.8%)
Housing situation:			
Rented from council	548 (22.1%)	316 (22.8%)	85 (5.0%)
Rented from private landlord	533 (21.5%)	409 (29.5%)	252 (14.9%)
Homeowner	1271 (51.3%)	587 (42.3%)	1158 (68.5%)
Other	126 (5.1%)	75 (5.4%)	195 (11.5%)
Region:			
North East	99 (4.0%)	95 (6.8%)	58 (3.4%)
North West	276 (11.0%)	123 (8.8%)	172 (10.2%)
Yorkshire and the humber	202 (8.1%)	103 (7.4%)	126 (7.4%)
East Midlands	178 (7.1%)	110 (7.9%)	123 (7.3%)
West Midlands	231 (9.2%)	131 (9.4%)	110 (6.5%)
East (Anglia)	223 (8.9%)	96 (6.9%)	154 (9.1%)
London	327 (13.1%)	215 (15.4%)	141 (8.3%)
South East	354 (14.2%)	184 (13.2%)	356 (21.0%)
South West	203 (8.1%)	88 (6.3%)	216 (12.8%)
Wales	209 (8.4%)	84 (6.0%)	96 (5.7%)
Scotland	125 (5.0%)	117 (8.4%)	120 (7.1%)
Northern Ireland	73 (2.9%)	53 (3.8%)	21 (1.2%)
Employment status:			
Unemployed	148 (5.9%)	113 (8.1%)	58 (3.4%)
Employed full-time	1098 (44.0%)	626 (44.8%)	339 (20.0%)
Employed part-time	387 (15.5%)	244 (17.5%)	219 (12.9%)
Self-employed	139 (5.6%)	95 (6.8%)	141 (8.3%)
Retired	360 (14.4%)	111 (7.9%)	515 (30.4%)
Student	90 (3.6%)	70 (5.0%)	308 (18.2%)
Homemaker	168 (6.7%)	76 (5.4%)	34 (2.0%)
Voluntary	3 (0.1%)	4 (0.3%)	28 (1.7%)
Disabled/Long-term sick leave	105 (4.2%)	59 (4.2%)	51 (3.0%)
Contact with mental health services in last 6 months			
Yes	553 (22.1%)	380 (27.2%)	369 (21.8%)
No	1948 (77.9%)	1019 (72.8%)	1324 (78.2%)
Current diagnosis of mental health problem			
Yes	658 (26.3%)	458 (32.8%)	413 (24.4%)
No	1842 (73.7%)	940 (67.2%)	1279 (75.6%)

The full item pool used to develop the scale is available in online Supplementary (Table S3.1). The Pearson correlation matrix was assessed for multi-collinearity (*r* < 0.95) and non-collinearity (*r* < 0.3), and three items (pool items 46, 52, and 77) were subsequently removed. Bartlett's test for Sphericity concluded that correlations between items were appropriate for EFA (χ^2^ = 118 120.3, df = 4095, *p* < 0.001). Sampling adequacy based on the KMO measure was at a superior level (overall KMO = 0.99, all items KMO>0.98). Parallel analysis showed that four or five-factor solutions appeared viable. Upon performing the EFA analysis, a four-factor solution emerged and was identified as the most appropriate model from a theoretical and empirical perspective. Iteratively running the EFA based on item removal criteria led to the retention of 46 items, with a further five items being removed due to ambiguous or repetitive item content (see online Supplementary Table S3.1). A CFA in the validation sample showed that a 41-item, 4 factor model was within the acceptable fit range [*N* = 1250; Robust χ^2^ = 3487.217, df = 773, *p* < 0.001, CFI = 0.916, TLI = 0.911, RMSEA = 0.053 (0.052–0.054), SRMSR = 0.039].

Four regSEM lasso were estimated with an iteration step of 0.03. The *λ* values for each regSEM lasso varied between 0 and 0.06 according to the final selected model with the lowest RMSEA. All items that had a coefficient of >0.02 were retained. The results showed that eight out of fourteen items were retained in the first factor, ten out of seventeen items were retained in the second factor, all five items were retained in the third factor, and four out of five items were retained in the fourth factor. Three additional items were removed after reviewing the item content qualitatively, resulting in a final set of 24 items with excellent model fit (*N* = 1250; robust χ^2^ = 995.676; df = 246; CFI = 0.956; TLI = 0.951; RMSEA = 0.049 (0.047–0.052); SRMR = 0.031). The factor loadings and factor correlations can be found in Tables 2 and 3.

**Table 2.** Confirmatory factor loadings based on 24 items (validation data)

Item	Loading	Factor
Item 1 – I can make a difference	0.65	F1 (mastery)
Item 3 – I am useful	0.83	
Item 7 – I have a purpose	0.79	
Item 10 – I can achieve things	0.86	
Item 12 – I can do things well	0.83	
Item 15 – I can succeed^a^	0.87	
Item 17 – I am worthwhile^a^	0.87	
Item 33 – I am strong	0.81	F2 (strength)
Item 37 – I can keep going	0.80	
Item 42 – I can succeed in challenging situations	0.85	
Item 45 – I can cope with anything	0.79	
Item 49 – I rise to the challenge^a^	0.86	
Item 51 – I don't give up	0.82	
Item 55 – I will be okay	0.81	
Item 57 – I can do things as well as anyone else^a^	0.82	
Item 62 – I can enjoy things	0.84	F3 (enjoyment)
Item 63 – I can relax^a^	0.82	
Item 64 – I can switch off	0.74	
Item 65 – I can have fun^a^	0.90	
Item 66 – I can do fun things	0.88	
Item 78 – I am reliable	0.77	F4 (character)
Item 80 – I am thoughtful	0.79	
Item 84 – I am a good person^a^	0.83	
Item 87 – I am helpful^a^	0.81	

a Items selected for the short-form version.

**Table 3:** **.** Factor correlations based on 24 items (validation data)

	f1 (mastery)	f2 (strength)	f3 (enjoyment)	f4 (character)
f2	0.89	1		
f3	0.75	0.76	1	
f4	0.73	0.76	0.73	1

Model fit results were considered satisfactory based on the additional data collected from the non-representative sample [*N* = 1399; Robust χ^2^ = 820.16, df = 246, *p* < 0.001, CFI = 0.969, TLI = 0.965, RMSEA = 0.041 (0.038–0.043), SRMSR = 0.027] and data collected via social media advertisement [*N* = 1693; Robust χ^2^ = 2385.533, df = 246, *p* < 0.001, CFI = 0.912, TLI = 0.901, RMSEA = 0.072 (0.069–0.074), SRMSR = 0.048]. The factor loadings and correlation of the additional CFAs can be found in Section 4 of online Supplementary materials.

Concerning gender and age measurement invariance, the configural invariance model achieved good model fit across all indices in both models (see online Supplementary Tables S5-1-2). Adding metric and scalar constraints only led to minute changes in fit and improvement in BIC, indicating that scalar invariance held across gender and age groups. Thus, the factor scores could be directly compared between age and gender groups. A comparison between gender groups revealed that the latent factor means for the first three factors were significantly higher for male participants than female participants (*F*_1_ = 0.153, adjusted-*p* = 0.032; *F*^2^ = 0.224, adjusted-*p* = 0.0004; *F*_3_ = 0.155, adjusted-*p* = 0.024). No significant differences were found for the fourth factor (*F*_4_ = −0.033, adjusted-*p* = 0.538). Latent factor means for older participants were found to be significantly higher across all factors (*F*_1_ = 0.194, adjusted-*p* < 0.001; *F*_2_ = 0.178, adjusted-*p* < 0.001; *F*_3_ = 0.252, adjusted-*p* < 0.001; *F*_4_ = 0.298, adjusted-*p* < 0.001).

A higher-order CFA model was carried out to evaluate the appropriateness of calculating a total scale score. The result showed excellent model fit [*N* = 1250; Robust χ^2^ = 1023.153, df = 248, *p* < 0.001, CFI = 0.955, TLI = 0.95, RMSEA = 0.05 (0.047–0.053), SRMSR = 0.034]. All subscales showed excellent omega coefficient: *F*_1_, *ω* = 0.93; *F*_2_, *ω* = 0.94; *F*_3_, *ω* = 0.92; *F*_4_, *ω* = 0.88. The overall internal consistency of the test was excellent (*ω* = 0.92). The one-week test–retest reliability (*N* = 659) for the subscales and overall scores were excellent: *F*_1_ = 0.88; *F*_2_ = 0.87; *F*_3_ = 0.82; *F*_4_ = 0.81; overall = 0.90).

Two items from each factor were selected to form an 8-item short-form measure. The goodness-of-fit for the correlated CFA model [*N* = 1250; robust χ^2^ = 44.078; df = 14; CFI = 0.993; TLI = 0.986; RMSEA = 0.041 (0.03–0.054); SRMR = 0.015] and higher order CFA model [*N* = 1250; robust χ^2^ = 56.936; df = 16; CFI = 0.990; TLI = 0.983; RMSEA = 0.045 (0.035–0.056); SRMR = 0.021] were excellent. The factor loadings, factor correlations and correlated residuals can be found in the online Supplementary materials (Section 6 online Supplementary Tables S6-1:3). The subscales and overall measure showed excellent internal consistency (*F*_1_, *ω* = 0.86; *F*_2_, *ω* = 0.83; *F*_3_, *ω* = 0.82; *F*_4_, *ω* = 0.81; overall, *ω* = 0.89). The total score of the short-form version had a correlation of *r* = 0.98 with the total score of the full 24-item version (explaining 96% of the variance in the full version). We also evaluated the 8-item measure based on the additional data. Correlated factor model fit results were considered excellent based on the non-representative sample [*N* = 1399; Robust χ^2^ = 23.804, df = 14, *p* < 0.048, CFI = 0.998, TLI = 0.995, RMSEA = 0.022 (0.008–0.035), SRMSR = 0.012] and data collected via social media advertisement [*N* = 1703; Robust χ^2^ = 89.039, df = 14, *p* < 0.001, CFI = 0.985, TLI = 0.971, RMSEA = 0.056 (0.047–0.066), SRMSR = 0.02]. The factor loadings and correlation of these additional CFAs can be found in online supplementary materials (online Supplementary Tables S6-3–S6-6).

For the long and short versions of the scale, separate SEM regressions showed that the individual factors were each predictive of well-being (see online Supplementary Table S7-1). For the 24-item version, regressing all four positive thoughts factors onto the well-being factor in a single SEM showed that only the first three factors were significant predictors with good model fit [*N* = 2500; robust χ^2^ = 3847.593; df = 656; CFI = 0.942; TLI = 0.938; RMSEA = 0.044 (0.043–0.045); SRMR = 0.032]. The fourth factor resulted in a negative coefficient, which was previously positive in the simple SEM regressions. Such a phenomenon was likely due to suppressor effects caused by shared variance among the predictors (Maassen & Bakker, 2001) and hence was removed using a backward elimination approach. The final SEM model with three significant predictors explained 68.6% of the variance in well-being (see online Supplementary Table S7–2). For the short-form, the model fit was excellent [*N* = 2500; robust χ^2^ = 1476.493; df = 201; CFI = 0.953; TLI = 0.946; RMSEA = 0.050 (0.048–0.052); SRMR = 0.048], but only the first and third factors were significant predictors of well-being (see Table 4), with 70% of the variance explained.

**Table 4:** **.** Correlations in the representative cohort (*N* = 2500) between the Oxford Positive Self Scale and the other assessments (using raw scores for all scales) (all correlations are significant at *p* < 0.001)

	Psychological well-being (WEMWBS)	Depression (PHQ-9)	Paranoia (R-GPTS Part A)	Paranoia (R-GPTS Part B)	Paranoia (total R-GPTS)	Hallucinations (SPEQ)	Social Comparison (SCS)	Self-Esteem (RSES)	Positive- Self (BCSS)	Negative-Self (BCSS)	Agoraphobia avoidance (O-AS)	Agoraphobia distress (O-AS)
24-item OxPos												
Total score	0.792	−0.487	−0.251	−0.202	−0.231	−0.200	0.715	0.672	0.697	−0.495	−0.319	−0.343
Factor 1	0.718	−0.441	−0.204	−0.151	−0.180	−0.154	0.668	0.636	0.661	−0.444	−0.261	−0.286
Factor 2	0.737	−0.429	−0.199	−0.147	−0.175	−0.140	0.665	0.622	0.649	−0.443	−0.273	−0.306
Factor 3	0.738	−0.482	−0.267	−0.232	−0.256	−0.213	0.640	0.587	0.587	−0.451	−0.325	−0.352
Factor 4	0.581	−0.366	−0.257	−0.233	−0.252	−0.261	0.526	0.503	0.542	−0.428	−0.301	−0.283
8-item OxPos												
Total score	0.775	−0.470	−0.243	−0.195	−0.223	−0.202	0.704	0.660	0.690	−0.487	−0.315	−0.337
Factor 1	0.699	−0.432	−0.19	−0.139	−0.167	−0.144	0.656	0.625	0.643	−0.436	−0.245	−0.281
Factor 2	0.677	−0.386	−0.169	−0.120	−0.146	−0.118	0.621	0.581	0.617	−0.398	−0.247	−0.284
Factor 3	0.713	−0.460	−0.254	−0.222	−0.244	−0.205	0.615	0.561	0.563	−0.434	−0.313	−0.337
Factor 4	0.560	−0.327	−0.223	−0.192	−0.213	−0.232	0.514	0.485	0.537	−0.403	−0.277	−0.250
Psychological well-being (WEMWBS)		−0.547	−0.250	−0.205	−0.232	−0.197	0.727	0.680	0.647	−0.510	−0.287	−0.328

As summarised in Table 4, the 24-item and 8-item OxPos scales had strong positive correlations with psychological well-being (WEMBS), positive social comparison (SCS), self-esteem (RSES), and positive self-beliefs (BCSS), moderate negative correlations with depression (PHQ-9), negative self-beliefs (BCSS), and agoraphobia (O-AS), and small negative correlations with paranoia (R-GPTS) and hallucinations (SPEQ).

In the representative group the mean score for the 24-item OxPos scale was 57.94 (s.d. = 21.26) (*N* = 2500) (mean scores by corresponding percentile scores are presented in Section 9 of online Supplementary materials). The mean score for the WEMWBS was 45.34 (s.d. = 12.16) (*N* = 2500). The correlation between the OxPos and WEMWBS was high (*r* = 0.79, *p* < 0.001). To determine cut-offs for the OxPos that will allow discrimination between participants who have a very low or a low level of positive self-beliefs in relation to psychological well-being from the rest of the population, we used the lowest 15 and 25% of population scorers on the WEMWBS (score = 33, score = 38 respectively), representing approximately 1.01 standard deviations and 0.6 standard deviations below the average score in the sample. ROC analysis identified 46 as an optimal cut-off point (sensitivity = 0.809; specificity = 0.835) with an overall AUC of 0.899 for the lowest 15% of the WEMWBS and 50 as an optimal cut-off point (sensitivity = 0.811; specificity = 0.811) with an overall AUC of 0.889 for the lowest 25% of the WEMWBS. A score of 46 on the Oxford Positive Self Scale would represent 0.56 standard deviations below the mean, which is the lowest 29% of the sample, and a score of 50 on the Positive Thoughts scale would represent 0.37 standard deviations below the mean, which is the lowest 36% of the sample.

In the representative group the mean score for the 8-item OxPos scale was 19.63 (s.d. = 7.4) (*N* = 2500) (mean scores by corresponding percentile scores are presented in Section 9 of online Supplementary materials). The correlation between the OxPos scale and the WEMWBS was moderately high (*r* = 0.77, *p* < 0.001). Given the small number of items in the OxPos short version, we only used the lowest 25% of scorers on the WEMWBS to determine the cut-off point. ROC analysis identified 16 as an optimal cut-off point (sensitivity = 0.857; specificity = 0.731) with an overall AUC of 0.882. A score of 16 on the OxPos short-form scale would represent approximately 0.49 standard deviations below the mean, which is the lowest 33% scorers in the sample. A summary of cut-offs including by age and gender are provided in Table 5 (with further detail provided in Sections 8 and 9 of the online Supplementary materials).

**Table 5:** **.** Cut-offs for the overall sample and by age and gender for the OxPos long and short-forms

	Long-form cut-off for the lowest 15% of WEMWBS scorers (sensitivity, specificity)	Long-form cut-off for the lowest 25% of WEMWBS scorers (sensitivity, specificity)	Short-form cut-off for the lowest 25% of WEMWBS scorers (sensitivity, specificity)
Overall sample	46 (0.809–0.835)	50 (0.811–0.811)	16 (0.857–0.731)
Male			
18–30 years old	36 (0.957–0.771)	53 (0.775–0.817)	16 (0.863–0.717)
31–50 years old	48 (0.759–0.836)	49 (0.787–0.730)	21 (0.580–0.900)
51+ years old	46 (0.896–0.827)	54 (0.855–0.814)	19 (0.848–0.779)
Female			
18–30 years old	42 (0.773–0.848)	46 (0.753–0.853)	15 (0.791–0.787)
31–50 years old	41 (0.828–0.795)	45 (0.827–0.762)	15 (0.846–0.713)
51+ years old	48 (0.833–0.894)	50 (0.875–0.830)	19 (0.820–0.877)

## Discussion

The Oxford Positive Self Scale is very clear for people to complete, assesses cognitions closely connected to psychological well-being, and has excellent reliability and validity established in a representative general population cohort. The four factors of the questionnaire link to established psychological intervention techniques. Beliefs about achieving things, doing things well, and succeeding relate to behavioural activation and mastery and control methods (Dimidjian, Barrera, Martell, Muñoz, & Lewinsohn, 2011). Beliefs about coping, not giving up, and keeping going relate to commonly used behavioural experiments in challenging situations (Bennett-Levy et al., 2004). Beliefs about enjoying things and being able to relax relate to savouring and relaxation techniques (Manzoni, Pagnini, Castelnuovo, & Molinari, 2008; Seligman, Steen, Park, & Peterson, 2005). Beliefs about being a good person relate to strengths and values identification (Seligman et al., 2005). Hence the scale may prove useful in assessing interventions to increase psychological well-being. There is also a short-form, explaining most of the variance in the full version, that could be used for weekly monitoring during intervention or in larger scale epidemiological research studies of well-being. It is also notable that the scale includes cut-offs by gender and age that can identify people who may benefit from an evidence-based intervention. Overall this is a scale likely to fit the experiences of those asked to complete it, provide direction for intervention, and enable precise assessment of the concept of positive self-beliefs.

There are limitations to the scale development. We used a non-probability online quota sampling method, which is a much stronger approach than most scale development studies that use convenience approaches, but it will have still introduced bias as to who was approached to take part. We do know that, taken as a whole, the respondents in this survey were broadly representative of the adult general population on a number of basic demographic features but not that individual respondents were representative of the general population. It is notable that the level of recent reported contact with mental health services was high, which may have affected cut-off scores. We used a large item pool, developed with people with lived experience of mental health difficulties, but the items included would not have been exhaustive, and therefore other types of important positive self-beliefs could well have been omitted. We would not claim that these are the only types of positive self-beliefs to target to improve psychological well-being. Nor do we know whether these cognitions are causal in improving well-being, which can only be determined via randomised controlled experiments or trials that intervene on the cognitions. At this stage we do not know whether the measure will be sensitive to change, although there is nothing to suggest in its face validity that this would not be the case, nor what the minimal clinically important change in the cognitions might be. Finally, although the representative sample would have included people with mental health problems and the Oxford Positive Self Scale correlated as expected with dimensional symptom measures, it would be valuable to obtain data on the scale from clinical cohorts in mental health services.

## Supporting information

Freeman et al. supplementary material 1 Freeman et al. supplementary material

Freeman et al. supplementary material 2 Freeman et al. supplementary material

## Supplementary material

The supplementary material for this article can be found at https://doi.org/10.1017/S0033291723000624.
