# Supplementary material for: The Oxford Positive Self Scale: psychometric development of an assessment of cognitions associated with psychological well-being
Source: Psychol Med. 2023 Mar 17;53(15):7161–9. doi: 10.1017/S0033291723000624 (PMC10719672; doi:10.1017/S0033291723000624)
Supplement: Freeman et al. supplementary material 1 — Freeman et al. supplementary material [file S0033291723000624sup001.docx]

**The Oxford Positive Self Scale**

This questionnaire lists beliefs that people can hold about themselves. Please indicate how strongly you hold each of the following beliefs by **circling a number** **0 (Do not believe it)** **– 4 (Believe it totally).**

Try to judge the beliefs on how you have viewed yourself ***over the past week***.

|  | Do not believe it | Believe it slightly | Believe it moderately | Believe it very much | Believe it totally |
| --- | --- | --- | --- | --- | --- |
| 1. I can make a difference | 0 | 1 | 2 | 3 | 4 |
| 2. I am useful | 0 | 1 | 2 | 3 | 4 |
| 3. I have a purpose | 0 | 1 | 2 | 3 | 4 |
| 4. I can achieve things | 0 | 1 | 2 | 3 | 4 |
| 5. I can do things well | 0 | 1 | 2 | 3 | 4 |
| 6. I can succeed | 0 | 1 | 2 | 3 | 4 |
| 7. I am worthwhile | 0 | 1 | 2 | 3 | 4 |
| 8. I am strong | 0 | 1 | 2 | 3 | 4 |
| 9. I can keep going | 0 | 1 | 2 | 3 | 4 |
| 10. I can succeed in challenging situations | 0 | 1 | 2 | 3 | 4 |
| 11. I can cope with anything | 0 | 1 | 2 | 3 | 4 |
| 12. I rise to the challenge | 0 | 1 | 2 | 3 | 4 |
| 13. I don’t give up | 0 | 1 | 2 | 3 | 4 |
|  | Do not believe it | Believe it slightly | Believe it moderately | Believe it very much | Believe it totally |
| 14. I will be okay | 0 | 1 | 2 | 3 | 4 |
| 15. I can do things as well as anyone else | 0 | 1 | 2 | 3 | 4 |
| 16. I can enjoy things | 0 | 1 | 2 | 3 | 4 |
| 17. I can relax | 0 | 1 | 2 | 3 | 4 |
| 18. I can switch off | 0 | 1 | 2 | 3 | 4 |
| 19. I can have fun | 0 | 1 | 2 | 3 | 4 |
| 20. I can do fun things | 0 | 1 | 2 | 3 | 4 |
| 21. I am reliable | 0 | 1 | 2 | 3 | 4 |
| 22. I am thoughtful | 0 | 1 | 2 | 3 | 4 |
| 23. I am a good person | 0 | 1 | 2 | 3 | 4 |
| 24. I am helpful | 0 | 1 | 2 | 3 | 4 |

**The Oxford Positive Self Scale Short Form**

This questionnaire lists beliefs that people can hold about themselves. Please indicate how strongly you hold each of the following beliefs by **circling a number** **0 (Do not believe it)** **– 4 (Believe it totally).**

Try to judge the beliefs on how you have viewed yourself ***over the past week***.

|  | Do not believe it | Believe it slightly | Believe it moderately | Believe it very much | Believe it totally |
| --- | --- | --- | --- | --- | --- |
| 1. I can succeed | 0 | 1 | 2 | 3 | 4 |
| 2. I am worthwhile | 0 | 1 | 2 | 3 | 4 |
| 3. I rise to the challenge | 0 | 1 | 2 | 3 | 4 |
| 4. I can do things as well as anyone else | 0 | 1 | 2 | 3 | 4 |
| 5. I can relax | 0 | 1 | 2 | 3 | 4 |
| 6. I can have fun | 0 | 1 | 2 | 3 | 4 |
| 7. I am a good person | 0 | 1 | 2 | 3 | 4 |
| 8. I am helpful | 0 | 1 | 2 | 3 | 4 |
