# Supplementary material for: The Oxford Positive Self Scale: psychometric development of an assessment of cognitions associated with psychological well-being
Source: Psychol Med. 2023 Mar 17;53(15):7161–9. doi: 10.1017/S0033291723000624 (PMC10719672; doi:10.1017/S0033291723000624)
Supplement: Freeman et al. supplementary material 2 — Freeman et al. supplementary material [file S0033291723000624sup002.docx]

**Supplementary materials**

Oxford Positive Self Scale – Original item pool.

| 1.     I can make a difference. |
| --- |
| 2.     I can get things done. |
| 3. I am useful. |
| 4.     I am capable. |
| 5.     I am successful. |
| 6.     I am talented. |
| 7.     I have a purpose. |
| 8.     I can get tasks done. |
| 9.     I can do things. |
| 10.   I can achieve things. |
| 11.   I can do good things. |
| 12.   I can do things well. |
| 13.   I can learn new things. |
| 14. I can master things. |
| 15.   I can succeed. |
| 16.   I can complete things. |
| 17.   I am worthwhile. |
| 18.   I am confident. |
| 19.   I am decisive. |
| 20.   I am determined. |
| 21.   I can do whatever I put my mind to. |
| 22.   I am happy with my decisions. |
| 23.   I achieve my goals. |
| 24.   I am a doer. |
| 25.   I work hard. |
| 26.   I am competent. |
| 27.   I finish what I start. |
| 28.   I am conscientious. |
| 29.   I care about what I do. |
| 30.   I like to do things as well as I can. |
| 31.   I give my all to achieve my goals. |
| 32.   I can do this. |
| 33.   I am strong. |
| 34.   I am brave. |
| 35.   I can try things. |
| 36.   I am resilient. |
| 37. I can keep going. |
| 38.   I am tough. |
| 39.   I can bounce back. |
| 40.   I am fearless. |
| 41. I stick at things. |
| 42.   I can succeed in challenging situations. |
| 43.   I am a winner. |
| 44. I have strengths. |
| 45.   I can cope with anything. |
| 46.   I can deal with anything. |
| 47. I can cope. |
| 48. I am a survivor. |
| 49. I rise to the challenge. |
| 50. Tough stuff doesn’t faze me. |
| 51. I don’t give up. |
| 52. I don’t quit. |
| 53. I keep trying. |
| 54. I don’t let things get to me. |
| 55. I will be okay. |
| 56. I can handle difficult tasks. |
| 57. I can do things as well as anyone else. |
| 58. I learn from my mistakes. |
| 59. I am in control. |
| 60. Things will work out fine. |
| 61. I’ve got this. |
| 62. I can enjoy things. |
| 63. I can relax. |
| 64. I can switch off. |
| 65. I can have fun. |
| 66. I can do fun things. |
| 67. I am a fun person. |
| 68. I am comfortable with myself. |
| 69. I am open to new experiences. |
| 70. I am open-minded. |
| 71. I am curious. |
| 72. I am interested in the world around me. |
| 73. I love life. |
| 74. I deserve good things. |
| 75. I am worthwhile. |
| 76. I am kind. |
| 77. I am honest. |
| 78. I am reliable. |
| 79. I am interesting. |
| 80. I am thoughtful. |
| 81. I have value. |
| 82. I am valuable. |
| 83. I am likeable. |
| 84. I am a good person. |
| 85. I like myself. |
| 86. I am creative. |
| 87. I am helpful. |
| 88. I belong. |
| 89. I have great ideas. |
| 90. I have people who believe in me. |
| 91. I have a lot to be proud of. |
| 92. I’m my own person. |
| 93. I know what matters to me. |
| 94. I am content. |

**Section 1**: Regularized Structural Equation Modelling (RegSEM)

Regularised structural equation modelling (RegSEM) is a statistical technique designed to assess SEMs using various forms of penalties to reduce the complexity and bias of the parameter estimates (Jacobucci, Grimm & McArdle, 2016). The regularisation method aims to improve model accuracy and reduce overfitting, with the most common regularisation methods being ridge regression, lasso, and elastic net. The penalty parameter determines the strength of the regularization, with a larger penalty resulting in more shrinkage and a smaller penalty resulting in less shrinkage of the parameter estimates. The optimal penalty value is often chosen using cross-validation or other model selection techniques and several model fit can be to assess the overall best fitting model.

We regressed the well-being factor on the item pool and only retained items with a regression coefficient > 0.02. We conducted regSEM separately for each factor so that the items from each factor were not overly penalised. The lasso regularization (Tibshirani, 1996) was employed to artificially drive the coefficient down towards zero via an exploratory search to simplify the measurement model and retain important predictors. A range of penalty values was evaluated to determine the best-fitting model based on the lowest Root Mean Square Error or Approximately (RMSEA) [Jacobucci, et al., 2016]. Only the coefficients of the best-fitting model were used to retain items.

**Section 2**: Measurement Invariance

The first step of measurement invariance (MI) was to employ a configural invariance model, where the CFA model is the same for both groups but the parameters are allowed to be freely estimated across groups. Achieving a good model fit indicates that both groups have the same factor structure. In the next step, we performed the weak/metric invariance model, where the factor loadings are constrained to be equal across groups. Assessing and comparing the model fit results to the preceding model will determine whether MI is achieved at this level. Next, we performed the strong/scalar invariance model, where the factor loadings and the item intercepts were constrained to be equal across groups, but the factor means were allowed to differ between groups. We can conclude that the item intercepts are equivalent across groups if it does not show a poorer fit than the weak/metric invariance model.

Given that the item intercepts are equivalent, the factor scores can be compared between groups and any differences in factor scores are not driven by specific items. In such instances, the latent factor means of the reference group are set to zero for model identification purposes, and the latent factor means for the other groups are freely estimated and compared to the reference group. The p-values between comparison groups were adjusted based on the Holm method (Aickin & Gensler, 1996; Holm, 1979).

We considered a constrained model to show poorer fit than the unconstrained or less constrained alternative based on the following criteria: For the weak/metric invariance model, a change of ≥ -.01 in CFI, a change of ≥ .015 in RMSEA or a change of ≥ .030 in SRMR would indicate a lack of invariance loadings. The cut-off criteria for scalar invariance testing are the same for CFI and RMSEA, but a change of ≥ .010 is recommended instead for SRMR (Chen, 2007). The Akaike Information Criterion (AIC) and the Bayesian Information Criterion (BIC) were also used to compare between nested models. Lower values for both indexes indicate better trade-offs between model fit and model complexity (van de Schoot, Lugtig, & Hox, 2012). The BIC is typically preferred when the indexes disagree in MI model selection (Anderson & Burnham, 2004).

**Section 3**: Exploratory Factor Analysis

| Table S3-1. Factor Loadings of EFA (n=46 items) | | | | |
| --- | --- | --- | --- | --- |
| Item | F1 | F2 | F3 | F4 |
| PT1_I can make a difference | 0.68 | NA | NA | NA |
| PT2_I can get things done | 0.82 | NA | NA | NA |
| PT3_I am useful | 0.80 | NA | NA | NA |
| PT4_I am capable | 0.78 | NA | NA | NA |
| PT5_I am successful | 0.54 | NA | NA | NA |
| PT6_I am talented | 0.52 | NA | NA | NA |
| PT7_I have a purpose | 0.70 | NA | NA | NA |
| PT8_I can get tasks done* | 0.85 | NA | NA | NA |
| PT9_I can do things* | 0.86 | NA | NA | NA |
| PT10_I can achieve things | 0.85 | NA | NA | NA |
| PT11_I can do good things | 0.83 | NA | NA | NA |
| PT12_I can do things well | 0.77 | NA | NA | NA |
| PT13_I can learn new things | 0.65 | NA | NA | NA |
| PT14_I can master things | 0.67 | NA | NA | NA |
| PT15_I can succeed | 0.63 | NA | NA | NA |
| PT16_I can complete things* | 0.75 | NA | NA | NA |
| PT17_I am worthwhile | 0.64 | NA | NA | NA |
| PT33_I am strong | NA | 0.66 | NA | NA |
| PT34_I am brave | NA | 0.78 | NA | NA |
| PT36_I am resilient* | NA | 0.59 | NA | NA |
| PT37_I can keep going | NA | 0.53 | NA | NA |
| PT38_I am tough | NA | 0.82 | NA | NA |
| PT39_I can bounce back | NA | 0.62 | NA | NA |
| PT40_I am fearless | NA | 0.82 | NA | NA |
| PT42_I can succeed in challenging situations | NA | 0.60 | NA | NA |
| PT43_I am a winner | NA | 0.68 | NA | NA |
| PT45_I can cope with anything | NA | 0.82 | NA | NA |
| PT47_I can cope* | NA | 0.62 | NA | NA |
| PT48_I am a survivor | NA | 0.59 | NA | NA |
| PT49_I rise to the challenge | NA | 0.60 | NA | NA |
| PT50_Tough stuff doesn't faze me | NA | 0.85 | NA | NA |
| PT51_I don't give up | NA | 0.55 | NA | NA |
| PT54_I don't let things get to me | NA | 0.75 | NA | NA |
| PT55_I will be okay | NA | 0.52 | NA | NA |
| PT56_I can handle difficult tasks | NA | 0.58 | NA | NA |
| PT57_I can do things as well as anyone else | NA | 0.53 | NA | NA |
| PT62_I can enjoy things | NA | NA | 0.65 | NA |
| PT63_I can relax | NA | NA | 0.84 | NA |
| PT64_I can switch off | NA | NA | 0.75 | NA |
| PT65_I can have fun | NA | NA | 0.72 | NA |
| PT66_I can do fun things | NA | NA | 0.69 | NA |
| PT76_I am kind | NA | NA | NA | 0.75 |
| PT78_I am reliable | NA | NA | NA | 0.60 |
| PT80_I am thoughtful | NA | NA | NA | 0.65 |
| PT84_I am a good person | NA | NA | NA | 0.68 |
| PT87_I am helpful | NA | NA | NA | 0.60 |
| *Item removed based on repetitive or unclear content | | | | |

**Section 4:** Confirmatory Factor Analysis

Table S4-1: Confirmatory factor loadings (41 items)

| Item | Estimate | SE | z-value | P-value | Std.lv | Std.all | Factor |
| --- | --- | --- | --- | --- | --- | --- | --- |
| PT1_I can make a difference | 1.00 |  |  |  | 0.75 | 0.66 | F1 |
| PT2_I can get things done | 1.13 | 0.04 | 30.44 | 0.00 | 0.85 | 0.77 |  |
| PT3_I am useful | 1.27 | 0.04 | 29.36 | 0.00 | 0.96 | 0.82 |  |
| PT4_I am capable | 1.24 | 0.04 | 28.07 | 0.00 | 0.93 | 0.82 |  |
| PT5_I am successful | 1.23 | 0.05 | 26.11 | 0.00 | 0.92 | 0.75 |  |
| PT6_I am talented | 1.15 | 0.04 | 27.18 | 0.00 | 0.86 | 0.71 |  |
| PT7_I have a purpose | 1.30 | 0.05 | 27.23 | 0.00 | 0.97 | 0.79 |  |
| PT10_I can achieve things | 1.37 | 0.05 | 27.68 | 0.00 | 1.03 | 0.87 |  |
| PT11_I can do good things | 1.20 | 0.05 | 25.11 | 0.00 | 0.90 | 0.81 |  |
| PT12_I can do things well | 1.28 | 0.05 | 26.36 | 0.00 | 0.96 | 0.85 |  |
| PT13_I can learn new things | 1.14 | 0.05 | 24.68 | 0.00 | 0.85 | 0.76 |  |
| PT14_I can master things | 1.25 | 0.05 | 25.53 | 0.00 | 0.94 | 0.80 |  |
| PT15_I can succeed | 1.36 | 0.05 | 27.09 | 0.00 | 1.02 | 0.85 |  |
| PT17_I am worthwhile | 1.43 | 0.05 | 26.25 | 0.00 | 1.07 | 0.84 |  |
| PT33_I am strong | 1.00 |  |  |  | 0.99 | 0.82 | F2 |
| PT34_I am brave | 0.96 | 0.02 | 46.97 | 0.00 | 0.96 | 0.77 |  |
| PT37_I can keep going | 0.90 | 0.02 | 37.76 | 0.00 | 0.89 | 0.80 |  |
| PT38_I am tough | 0.95 | 0.03 | 37.96 | 0.00 | 0.94 | 0.78 |  |
| PT39_I can bounce back | 0.95 | 0.02 | 40.62 | 0.00 | 0.94 | 0.83 |  |
| PT40_I am fearless | 0.92 | 0.03 | 32.31 | 0.00 | 0.92 | 0.73 |  |
| PT42_I can succeed in challenging situations | 0.96 | 0.02 | 40.09 | 0.00 | 0.96 | 0.85 |  |
| PT43_I am a winner | 1.03 | 0.03 | 39.24 | 0.00 | 1.03 | 0.81 |  |
| PT45_I can cope with anything | 0.99 | 0.03 | 38.41 | 0.00 | 0.98 | 0.81 |  |
| PT48_I am a survivor | 0.89 | 0.03 | 32.87 | 0.00 | 0.88 | 0.75 |  |
| PT49_I rise to the challenge | 1.02 | 0.03 | 40.12 | 0.00 | 1.01 | 0.86 |  |
| PT50_Tough stuff doesn't faze me | 1.01 | 0.03 | 37.62 | 0.00 | 1.01 | 0.82 |  |
| PT51_I don't give up | 0.96 | 0.03 | 37.54 | 0.00 | 0.95 | 0.81 |  |
| PT54_I don't let things get to me | 0.99 | 0.03 | 32.86 | 0.00 | 0.98 | 0.75 |  |
| PT55_I will be okay | 0.96 | 0.02 | 39.71 | 0.00 | 0.96 | 0.80 |  |
| PT56_I can handle difficult tasks | 0.96 | 0.03 | 38.52 | 0.00 | 0.96 | 0.85 |  |
| PT57_I can do things as well as anyone else | 1.00 | 0.03 | 39.48 | 0.00 | 0.99 | 0.81 |  |
| PT62_I can enjoy things | 1.00 |  |  |  | 0.94 | 0.84 | F3 |
| PT63_I can relax | 1.03 | 0.03 | 38.01 | 0.00 | 0.97 | 0.81 |  |
| PT64_I can switch off | 1.02 | 0.03 | 30.03 | 0.00 | 0.96 | 0.74 |  |
| PT65_I can have fun | 1.11 | 0.03 | 40.75 | 0.00 | 1.04 | 0.90 |  |
| PT66_I can do fun things | 1.12 | 0.03 | 38.91 | 0.00 | 1.05 | 0.88 |  |
| PT76_I am kind | 1.00 |  |  |  | 0.87 | 0.81 | F4 |
| PT78_I am reliable | 0.96 | 0.03 | 29.87 | 0.00 | 0.83 | 0.76 |  |
| PT80_I am thoughtful | 0.99 | 0.03 | 30.71 | 0.00 | 0.86 | 0.81 |  |
| PT84_I am a good person | 1.06 | 0.03 | 32.37 | 0.00 | 0.92 | 0.83 |  |
| PT87_I am helpful | 1.05 | 0.03 | 33.40 | 0.00 | 0.91 | 0.84 |  |
| Note: SE = Standard Error; Std.lv = Standardised latent variables; Std.all = Standardised both latent and observed variables | | | | | | | |

| Table S4-2. Confirmatory factor loadings based on 24 items (non-representative samples) | | | | | | | |
| --- | --- | --- | --- | --- | --- | --- | --- |
| Item | Estimate | SE | z-value | P(>\|z\|) | Std.lv | Std.all | Factor |
| PT17_I am worthwhile | 1.00 |  |  |  | 1.05 | 0.84 | F1 |
| PT15_I can succeed | 1.02 | 0.02 | 53.53 | 0.00 | 1.08 | 0.86 |  |
| PT3_I am useful | 0.89 | 0.02 | 40.99 | 0.00 | 0.94 | 0.78 |  |
| PT10_I can achieve things | 0.97 | 0.02 | 47.91 | 0.00 | 1.02 | 0.86 |  |
| PT12_I can do things well | 0.93 | 0.02 | 49.78 | 0.00 | 0.98 | 0.85 |  |
| PT1_I can make a difference | 0.70 | 0.03 | 26.45 | 0.00 | 0.73 | 0.63 |  |
| PT7_I have a purpose | 0.93 | 0.02 | 44.25 | 0.00 | 0.98 | 0.78 |  |
| PT55_I will be okay | 1.00 |  |  |  | 0.98 | 0.80 | F2 |
| PT45_I can cope with anything | 1.04 | 0.03 | 40.64 | 0.00 | 1.02 | 0.81 |  |
| PT33_I am strong | 1.04 | 0.03 | 41.45 | 0.00 | 1.02 | 0.81 |  |
| PT57_I can do things as well as anyone else | 1.04 | 0.02 | 45.03 | 0.00 | 1.02 | 0.82 |  |
| PT51_I don't give up | 1.02 | 0.03 | 41.26 | 0.00 | 0.99 | 0.80 |  |
| PT37_I can keep going | 0.98 | 0.02 | 41.60 | 0.00 | 0.97 | 0.81 |  |
| PT49_I rise to the challenge | 1.05 | 0.02 | 43.07 | 0.00 | 1.03 | 0.86 |  |
| PT42_I can succeed in challenging situations | 1.01 | 0.02 | 41.58 | 0.00 | 0.99 | 0.83 |  |
| PT64_I can switch off | 1.00 |  |  |  | 1.00 | 0.74 | F3 |
| PT62_I can enjoy things | 0.97 | 0.03 | 35.26 | 0.00 | 0.96 | 0.84 |  |
| PT63_I can relax | 1.05 | 0.02 | 51.83 | 0.00 | 1.05 | 0.82 |  |
| PT65_I can have fun | 1.04 | 0.03 | 33.41 | 0.00 | 1.04 | 0.86 |  |
| PT66_I can do fun things | 1.02 | 0.03 | 31.25 | 0.00 | 1.01 | 0.86 |  |
| PT87_I am helpful | 1.00 |  |  |  | 0.91 | 0.79 | F4 |
| PT78_I am reliable | 0.99 | 0.03 | 31.77 | 0.00 | 0.90 | 0.80 |  |
| PT84_I am a good person | 0.99 | 0.03 | 34.98 | 0.00 | 0.90 | 0.79 |  |
| PT80_I am thoughtful | 0.96 | 0.03 | 29.35 | 0.00 | 0.87 | 0.78 |  |
| Note: SE = Standard Error; Std.lv = Standardised latent variables; Std.all = Standardised both latent and observed variables | | | | | | | |

| Table S4-3. Inter-factor correlations based on 24 items (non-representative data) | | | | |
| --- | --- | --- | --- | --- |
|  | f1 | f2 | f3 | f4 |
| f1 | 1 |  |  |  |
| f2 | 0.89 | 1 |  |  |
| f3 | 0.78 | 0.81 | 1 |  |
| f4 | 0.7 | 0.73 | 0.73 | 1 |

| Table S4-4. Confirmatory factor loadings based on 24 items (data collected via Facebook advertisement) | | | | | | | |  |
| --- | --- | --- | --- | --- | --- | --- | --- | --- |
| Item | Estimate | SE | z-value | P-value | Std.lv | Std.all | Factor | |
| PT17_I am worthwhile | 1.00 |  |  |  | 1.07 | 0.85 | F1 | |
| PT15_I can succeed | 0.86 | 0.02 | 46.69 | 0.00 | 0.92 | 0.84 |  |  |
| PT3_I am useful | 0.89 | 0.02 | 54.24 | 0.00 | 0.96 | 0.85 |  |  |
| PT10_I can achieve things | 0.87 | 0.02 | 46.24 | 0.00 | 0.93 | 0.85 |  |  |
| PT12_I can do things well | 0.74 | 0.02 | 38.52 | 0.00 | 0.79 | 0.78 |  |  |
| PT1_I can make a difference | 0.72 | 0.02 | 35.04 | 0.00 | 0.78 | 0.71 |  |  |
| PT7_I have a purpose | 0.89 | 0.02 | 43.95 | 0.00 | 0.95 | 0.75 |  |  |
| PT55_I will be okay | 1.00 |  |  |  | 0.90 | 0.74 | F2 | |
| PT45_I can cope with anything | 0.97 | 0.03 | 35.71 | 0.00 | 0.88 | 0.74 |  |  |
| PT33_I am strong | 1.08 | 0.03 | 39.95 | 0.00 | 0.97 | 0.80 |  |  |
| PT57_I can do things as well as anyone else | 0.94 | 0.03 | 31.78 | 0.00 | 0.85 | 0.69 |  |  |
| PT51_I don't give up | 0.95 | 0.03 | 31.99 | 0.00 | 0.86 | 0.75 |  |  |
| PT37_I can keep going | 1.00 | 0.03 | 39.26 | 0.00 | 0.90 | 0.79 |  |  |
| PT49_I rise to the challenge | 1.04 | 0.03 | 36.84 | 0.00 | 0.94 | 0.84 |  |  |
| PT42_I can succeed in challenging situations | 0.98 | 0.03 | 34.96 | 0.00 | 0.88 | 0.82 |  |  |
| PT64_I can switch off | 1.00 |  |  |  | 0.93 | 0.73 | F3 | |
| PT62_I can enjoy things | 1.05 | 0.03 | 37.47 | 0.00 | 0.98 | 0.86 |  |  |
| PT63_I can relax | 1.03 | 0.02 | 60.30 | 0.00 | 0.96 | 0.76 |  |  |
| PT65_I can have fun | 1.18 | 0.04 | 32.80 | 0.00 | 1.10 | 0.94 |  |  |
| PT66_I can do fun things | 1.12 | 0.04 | 29.21 | 0.00 | 1.04 | 0.91 |  |  |
| PT87_I am helpful | 1.00 |  |  |  | 0.84 | 0.83 | F4 | |
| PT78_I am reliable | 0.91 | 0.03 | 30.19 | 0.00 | 0.77 | 0.72 |  |  |
| PT84_I am a good person | 1.08 | 0.03 | 36.79 | 0.00 | 0.90 | 0.82 |  |  |
| PT80_I am thoughtful | 0.89 | 0.03 | 34.41 | 0.00 | 0.75 | 0.75 |  |  |
| Note: SE = Standard Error; Std.lv = Standardised latent variables; Std.all = Standardised both latent and observed variables | | | | | | | |  |

| Table S4-5. Inter-factor correlations based on 24 items (data collected via Facebook advertisement) | | | | |
| --- | --- | --- | --- | --- |
|  | f1 | f2 | f3 | f4 |
| f1 | 1 |  |  |  |
| f2 | 0.88 | 1 |  |  |
| f3 | 0.69 | 0.63 | 1 |  |
| f4 | 0.72 | 0.75 | 0.57 | 1 |

**Section 5**: Measurement invariance

| Table S5-1. Model comparison to determine measurement invariance between gender groups (Male [n = 626] vs Female [n=621]) | | | | | | | |
| --- | --- | --- | --- | --- | --- | --- | --- |
| Model | Model Comparison | X2 | DF | RMSEA | SRMR | CFI | BIC |
| Configural | - | 1792.7 | 492 | 0.06 | 0.34 | 0.957 | 70808 |
| Weak/Metric | Configural vs Metric | 1807.5 | 512 | 0.058 (Δ = -0.002) | 0.36 (Δ = 0.002) | 0.957 (Δ = 0) | 70680 |
| Strong/Scalar | Metric vs Scalar | 1870.1 | 532 | 0.058 (Δ = 0) | 0.036 (Δ = 0) | .955 (Δ = -0.002) | 70600 |
| Note: DF = Degrees of Freedom; RMSEA = Root Mean Square Error of Approximation; SRMR = Standardised Root Mean Square Residual; CFI = Comparative Fit Index. | | | | | | | |

| Table S5-2. Model comparison to determine measurement invariance between Age groups (< 46 [n = 609] vs >= 46 [n=638]) | | | | | | | | |
| --- | --- | --- | --- | --- | --- | --- | --- | --- |
| Model | Model Comparison | X2 | DF | RMSEA | SRMR | CFI | BIC |  |
| Configural | - | 1922.3 | 492 | 0.064 | 0.034 | 0.952 | 70027 |  |
| Weak/Metric | Configural vs Metric | 1942.1 | 512 | 0.063 (Δ = -0.001) | 0.038 (Δ = 0.004) | 0.952 | 69905 |  |
| Strong/Scalar | Metric vs Scalar | 2071.7 | 532 | 0.064 (Δ = 0.001) | 0.039 (Δ = 0.001) | 0.948 (Δ = -0.004) | 69892 |  |
| Note: DF = Degrees of Freedom; RMSEA = Root Mean Square Error of Approximation; SRMR = Standardised Root Mean Square Residual; CFI = Comparative Fit Index. | | | | | | | | |

**Section 6**: Factor loadings of 8-item Short Form Oxford OxPosSelf Scale

| Table S6-1. Confirmatory factor loadings based on short form version (validation sample) | | | | | | | | |
| --- | --- | --- | --- | --- | --- | --- | --- | --- |
| Item | Estimate | SE | z-value | P-value | Std.lv | Std.all | Factor |  |
| PosThoughts17 | 1.00 |  |  |  | 1.10 | 0.88 | F1 |  |
| PosThoughts15 | 0.93 | 0.02 | 42.38 | 0.00 | 1.03 | 0.85 |  |  |
| PosThoughts57 | 1.00 |  |  |  | 1.02 | 0.83 | F2 |  |
| PosThoughts49 | 0.97 | 0.03 | 36.35 | 0.00 | 0.99 | 0.85 |  |  |
| PosThoughts63 | 1.00 |  |  |  | 0.99 | 0.82 | F3 |  |
| PosThoughts65 | 0.98 | 0.03 | 33.13 | 0.00 | 0.98 | 0.84 |  |  |
| PosThoughts87 | 1.00 |  |  |  | 0.90 | 0.82 | F4 |  |
| PosThoughts84 | 1.02 | 0.04 | 26.88 | 0.00 | 0.91 | 0.83 |  |  |
| Note: SE = Standard Error; Std.lv = Standardised latent variables; Std.all = Standardised both latent and observed variables | | | | | | | | |

| Table S6-2. Inter-factor correlations based on short form version (validation sample) | | | | |
| --- | --- | --- | --- | --- |
|  | f1 | f2 | f3 | f4 |
| f1 | 1 |  |  |  |
| f2 | 0.91 | 1 |  |  |
| f3 | 0.80 | 0.79 | 1 |  |
| f4 | 0.74 | 0.76 | 0.73 | 1 |
|  | | | | |

| Table. S6-3 Correlated Residuals based on short form version (validation sample) | | | | | | | | |
| --- | --- | --- | --- | --- | --- | --- | --- | --- |
|  | PT17 | PT15 | PT57 | PT49 | PT63 | PT65 | PT87 | PT84 |
| PosThoughts17 | 0.00 |  |  |  |  |  |  |  |
| PosThoughts15 | 0.00 | 0.00 |  |  |  |  |  |  |
| PosThoughts57 | 0.00 | 0.02 | 0.00 |  |  |  |  |  |
| PosThoughts49 | -0.02 | 0.01 | 0.00 | 0.00 |  |  |  |  |
| PosThoughts63 | 0.02 | -0.01 | 0.02 | 0.01 | 0.00 |  |  |  |
| PosThoughts65 | 0.02 | -0.04 | -0.01 | -0.02 | 0.00 | 0.00 |  |  |
| PosThoughts87 | 0.00 | -0.02 | -0.02 | 0.04 | -0.02 | 0.01 | 0.00 |  |
| PosThoughts84 | 0.02 | 0.00 | -0.03 | 0.01 | -0.02 | 0.02 | 0.00 | 0.00 |
|  | | | | | | | | |

| Table S6-4. Confirmatory factor loadings based on short form version (non-representative sample) | | | | | | | | |
| --- | --- | --- | --- | --- | --- | --- | --- | --- |
| Item | Estimate | Std.Err | z-value | P-value | Std.lv | Std.all | Factor | |
| PosThoughts17 | 1.00 |  |  |  | 1.06 | 0.85 | F1 | |
| PosThoughts15 | 1.00 | 0.02 | 44.37 | 0.00 | 1.07 | 0.85 |  |  |
| PosThoughts57 | 1.00 |  |  |  | 1.04 | 0.84 | F2 | |
| PosThoughts49 | 0.96 | 0.02 | 41.60 | 0.00 | 1.00 | 0.84 |  |  |
| PosThoughts63 | 1.00 |  |  |  | 1.03 | 0.81 | F3 | |
| PosThoughts65 | 0.97 | 0.03 | 34.61 | 0.00 | 1.00 | 0.83 |  |  |
| PosThoughts87 | 1.00 |  |  |  | 0.95 | 0.83 | F4 | |
| PosThoughts84 | 0.92 | 0.03 | 27.11 | 0.00 | 0.87 | 0.77 |  |  |
| Note: Std.Err = Standard Error; Std.lv = Standardised latent variables; Std.all = Standardised both latent and observed variables | | | | | | | |  |

| Table S6-5. Inter-factor correlations based on short form version (non-representative sample) | | | | |
| --- | --- | --- | --- | --- |
|  | f1 | f2 | f3 | f4 |
| f1 | 1 |  |  |  |
| f2 | 0.90 | 1 |  |  |
| f3 | 0.80 | 0.82 | 1 |  |
| f4 | 0.70 | 0.73 | 0.74 | 1 |
|  |  |  |  |  |

| Table. S6-6 Correlated Residuals based on short form version (non-representative sample) | | | | | | | | | |
| --- | --- | --- | --- | --- | --- | --- | --- | --- | --- |
|  | PT17 | PT15 | PT57 | PT49 | PT63 | PT65 | PT87 | PT84 |  |
| PosThoughts17 | 0.00 |  |  |  |  |  |  |  |  |
| PosThoughts15 | 0.00 | 0.00 |  |  |  |  |  |  |  |
| PosThoughts57 | -0.01 | 0.00 | 0.00 |  |  |  |  |  |  |
| PosThoughts49 | -0.01 | 0.02 | 0.00 | 0.00 |  |  |  |  |  |
| PosThoughts63 | 0.02 | 0.01 | 0.01 | 0.00 | 0.00 |  |  |  |  |
| PosThoughts65 | 0.00 | -0.02 | -0.01 | 0.00 | 0.00 | 0.00 |  |  |  |
| PosThoughts87 | 0.03 | -0.02 | 0.01 | -0.01 | -0.02 | 0.01 | 0.00 |  |  |
| PosThoughts84 | 0.01 | -0.02 | 0.00 | 0.00 | -0.02 | 0.02 | 0.00 | 0.00 |  |
|  | | | | | | | | | |

| Table S6-7. Confirmatory factor loadings based on short form version (Facebook sample) | | | | | | |  |  |
| --- | --- | --- | --- | --- | --- | --- | --- | --- |
| Item | Estimate | Std.Err | z-value | P-value | Std.lv | Std.all | Factor |  |
| PosThoughts17 | 1.00 |  |  |  | 1.08 | 0.86 | F1 |  |
| PosThoughts15 | 0.83 | 0.02 | 41.13 | 0.00 | 0.90 | 0.81 |  |  |
| PosThoughts57 | 1.00 |  |  |  | 0.88 | 0.72 | F2 |  |
| PosThoughts49 | 1.01 | 0.04 | 29.29 | 0.00 | 0.89 | 0.79 |  |  |
| PosThoughts63 | 1.00 |  |  |  | 1.00 | 0.80 | F3 |  |
| PosThoughts65 | 1.00 | 0.03 | 31.86 | 0.00 | 1.00 | 0.86 |  |  |
| PosThoughts87 | 1.00 |  |  |  | 0.83 | 0.81 | F4 |  |
| PosThoughts84 | 1.10 | 0.04 | 29.68 | 0.00 | 0.91 | 0.83 |  |  |
| Note: Std.Err = Standard Error; Std.lv = Standardised latent variables; Std.all = Standardised both latent and observed variables | | | | | | | | |

| Table S6-8. Inter-factor correlations based on short form version (Facebook sample) | | | | |
| --- | --- | --- | --- | --- |
|  | f1 | f2 | f3 | f4 |
| f1 | 1 |  |  |  |
| f2 | 0.91 | 1 |  |  |
| f3 | 0.78 | 0.65 | 1 |  |
| f4 | 0.74 | 0.79 | 0.61 | 1 |
|  |  |  |  |  |

| Table. S6-9 Correlated Residuals based on short form version (Facebook sample) | | | | | | | | | |
| --- | --- | --- | --- | --- | --- | --- | --- | --- | --- |
|  | PT17 | PT15 | PT57 | PT49 | PT63 | PT65 | PT87 | PT84 |  |
| PosThoughts17 | 0.00 |  |  |  |  |  |  |  |  |
| PosThoughts15 | 0.00 | 0.00 |  |  |  |  |  |  |  |
| PosThoughts57 | -0.02 | 0.05 | 0.00 |  |  |  |  |  |  |
| PosThoughts49 | -0.03 | 0.03 | 0.00 | 0.00 |  |  |  |  |  |
| PosThoughts63 | 0.03 | -0.03 | 0.02 | -0.03 | 0.00 |  |  |  |  |
| PosThoughts65 | 0.02 | -0.03 | 0.02 | 0.00 | 0.00 | 0.00 |  |  |  |
| PosThoughts87 | 0.01 | -0.04 | -0.03 | 0.03 | -0.02 | 0.02 | 0.00 |  |  |
| PosThoughts84 | 0.04 | -0.02 | -0.01 | -0.01 | -0.01 | 0.01 | 0.00 | 0.00 |  |
|  | | | | | | | | | |

**Section 7**: Structural Equation Modelling (SEM)

| Table S7-1. Simple SEM regressions between OxPos factors and well-being | | | | | | |
| --- | --- | --- | --- | --- | --- | --- |
| Factor | Unstandardised Beta | Standardised Beta | Adjusted p-value | R2 | Version |  |
| F1 | 0.55 | 0.76 | <0.001 | 0.58 | 26 items |  |
| F2 | 0.63 | 0.77 | <0.001 | 0.60 |  |  |
| F3 | 0.62 | 0.78 | <0.001 | 0.60 |  |  |
| F4 | 0.55 | 0.63 | <0.001 | 0.39 |  |  |
| F1 | 0.55 | 0.78 | <0.001 | 0.61 | 8 items |  |
| F2 | 0.59 | 0.76 | <0.001 | 0.59 |  |  |
| F3 | 0.63 | 0.81 | <0.001 | 0.65 |  |  |
| F4 | 0.54 | 0.62 | <0.001 | 0.39 |  |  |
| Note: Corrected for multiple comparisons using the Holm method. | | | | | | |

| Table S7-2. Predicting Well-being by OxPos factors based on SEM (short and long form) | | | | | | |
| --- | --- | --- | --- | --- | --- | --- |
| Factor | Unstandardised Beta Estimate | SE | Adjusted P-value | Standardised Beta estimate | Version |  |
| F1 | 0.17 | 0.03 | 0.00 | 0.23 | 26 items |  |
| F2 | 0.21 | 0.04 | 0.00 | 0.26 |  |  |
| F3 | 0.33 | 0.02 | 0.00 | 0.41 |  |  |
| F1 | 0.29 | 0.03 | 0.00 | 0.40 | 8 items |  |
| F3 | 0.39 | 0.03 | 0.00 | 0.49 |  |  |
| Note. SE = Standard Error | | | | | | |

**Section 8**. Creating Cut-offs

ROC curve analysis evaluates the relationship between the sensitivity (true positives) and the inverse of the specificity (true negatives) at each value along a screening scale based on two groups of interest (e.g. depressed vs non-depressed groups). The outcome variable is the area under the ROC curve (AUC) (Bradley, 1997). The AUC is defined as the probability that a randomly sampled participant will be correctly assigned to the targeted group (Hanley & McNeil, 1982). In other words, the AUC represents the overall accuracy of the scale to screen individuals and assign them to the appropriate group given their trait levels. The value of AUC ranges between zero and 1, where 0.5 indicates random performance and one indicates perfect performance. More specifically, 0.50 to 0.6 is an indication of unacceptably poor accuracy; 0.6-0.7 poor accuracy; 0.7-0.8 fair accuracy; 0.8-0.9 good accuracy (Metz, 1978).

*Proposed cut-offs*

*Overall representative data*

The mean score for the 8-item OxPos scale is 19.63 (SD = 7.4) and the mean score of WEMWBS is 45.34 (SD = 12.16), with N = 2500. The correlation between the OxPos scale and WEMWBS was moderately high (r = 0.77, p<0). Given the small number of items in the short version, we only used the bottom 25% of the WEMWBS to determine the cut-off point for the short form OxPos scale. ROC analysis identified 16 as an optimal cut-off point (sensitivity = 0.857; specificity = 0.731) with an overall AUC of 0.882. A score of 16 on the OxPos scale would represent approximately 0.49 SD below the mean, which is the bottom 33% of the sample.

*Male between 18 – 30*

The mean score for the 24-item OxPos scale is 57.45 (SD = 20.27) and the mean score of WEMWBS is 44.65 (SD = 12.57), with N = 242. The correlation between the OxPos scale and WEMWBS was moderately high (r = 0.73, p<0). To determine cut-offs for the OxPos scale that will allow us to discriminate participants who have very low or low positive thoughts from those who have moderate or high positive thoughts, we used the bottom 15% and 25% of the WEMWBS (score = 33, score = 38), representing approximately 0.93 SD and 0.53 SD below the average score in the sample.

ROC analysis identified 36 as an optimal cut-off point (sensitivity = 0.957; specificity = 0.771) with an overall AUC of 0.933 for the bottom 15% of the WEMWBS and 53 as an optimal cut-off point (sensitivity = 0.775; specificity = 0.817) with an overall AUC of 0.872 for the bottom 25% of the WEMWBS. A score of 36 on the OxPos scale would represent 1.06 SD below the mean, which is the bottom 16% of the sample, and a score of 53 on the OxPos scale would represent 0.22 SD below the mean, which is the bottom 42% of the sample.

The mean score for the 8-item OxPos scale is 19.3 (SD = 7.27) and the mean score of WEMWBS is 44.65 (SD = 12.57), with N = 242. The correlation between the OxPos scale and WEMWBS was moderately high (r = 0.7, p<0). Given the small number of items in the short version, we only used the bottom 25% of the WEMWBS to determine the cut-off point for the short form OxPosOxPos scale. ROC analysis identified 16 as an optimal cut-off point (sensitivity = 0.863; specificity = 0.717) with an overall AUC of 0.854. A score of 16 on the OxPos scale would represent approximately 0.45 SD below the mean, which is the bottom 33% of the sample.

*Male between 31 – 50*

The mean score for the 24-item OxPos scale is 57.97 (SD = 21.64) and the mean score of WEMWBS is 44.55 (SD = 12.33), with N = 400. The correlation between the OxPos scale and WEMWBS was moderately high (r = 0.75, p<0). To determine cut-offs for the OxPos scale that will allow us to discriminate participants who have very low or low positive thoughts from those who have moderate or high positive thoughts, we used the bottom 15% and 25% of the WEMWBS (score = 32, score = 37), representing approximately 1.02 SD and 0.63 SD below the average score in the sample.

ROC analysis identified 48 as an optimal cut-off point (sensitivity = 0.759; specificity = 0.836) with an overall AUC of 0.88 for the bottom 15% of the WEMWBS and 49 as an optimal cut-off point (sensitivity = 0.787; specificity = 0.73) with an overall AUC of 0.85 for the bottom 25% of the WEMWBS. A score of 48 on the OxPoss scale would represent 0.46 SD below the mean, which is the bottom 34% of the sample, and a score of 49 on the OxPos scale would represent 0.41 SD below the mean, which is the bottom 35% of the sample.

The mean score for the 8-item OxPos scale is 19.51 (SD = 7.53) and the mean score of WEMWBS is 44.55 (SD = 12.33), with N = 400. The correlation between the OxPos scale and WEMWBS was moderately high (r = 0.73, p<0). Given the small number of items in the short version, we only used the bottom 25% of the WEMWBS to determine the cut-off point for the short form OxPos scale. ROC analysis identified 21 as an optimal cut-off point (sensitivity = 0.58; specificity = 0.9) with an overall AUC of 0.833. A score of 21 on the OxPos scale would represent approximately -0.2 SD below the mean, which is the bottom 58% of the sample.

*Male greater than 51*

The mean score for the 24-item OxPos scale is 62.47 (SD = 20.33) and the mean score of WEMWBS is 47.92 (SD = 11.99), with N = 562. The correlation between the OxPos scale and WEMWBS was moderately high (r = 0.84, p<0). To determine cut-offs for the OxPos scale that will allow us to discriminate participants who have very low or low positive thoughts from those who have moderate or high positive thoughts, we used the bottom 15% and 25% of the WEMWBS (score = 36, score = 41), representing approximately 0.99 SD and 0.58 SD below the average score in the sample.

ROC analysis identified 46 as an optimal cut-off point (sensitivity = 0.896; specificity = 0.827) with an overall AUC of 0.918 for the bottom 15% of the WEMWBS and 54 as an optimal cut-off point (sensitivity = 0.855; specificity = 0.814) with an overall AUC of 0.904 for the bottom 25% of the WEMWBS. A score of 46 on the OxPos scale would represent 0.81 SD below the mean, which is the bottom 22% of the sample, and a score of 54 on the OxPos scale would represent 0.42 SD below the mean, which is the bottom 33% of the sample.

The mean score for the 8-item OxPos scale is 21.23 (SD = 7) and the mean score of WEMWBS is 47.92 (SD = 11.99), with N = 562. The correlation between the OxPos scale and WEMWBS was moderately high (r = 0.82, p<0). Given the small number of items in the short version, we only used the bottom 25% of the WEMWBS to determine the cut-off point for the short form OxPos scale. ROC analysis identified 19 as an optimal cut-off point (sensitivity = 0.848; specificity = 0.779) with an overall AUC of 0.895. A score of 19 on the OxPos scale would represent approximately 0.32 SD below the mean, which is the bottom 37% of the sample.

*Female between 18 – 30*

The mean score for the 24-item OxPos scale is 51.29 (SD = 20.53) and the mean score of WEMWBS is 42.3 (SD = 11.34), with N = 310. The correlation between the OxPos scale and WEMWBS was moderately high (r = 0.73, p<0). To determine cut-offs for the OxPos scale that will allow us to discriminate participants who have very low or low positive thoughts from those who have moderate or high positive thoughts, we used the bottom 15% and 25% of the WEMWBS (score = 30, score = 36), representing approximately 1.08 SD and 0.56 SD below the average score in the sample.

ROC analysis identified 42 as an optimal cut-off point (sensitivity = 0.773; specificity = 0.848) with an overall AUC of 0.884 for the bottom 15% of the WEMWBS and 46 as an optimal cut-off point (sensitivity = 0.753; specificity = 0.853) with an overall AUC of 0.884 for the bottom 25% of the WEMWBS. A score of 42 on the OxPos scale would represent 0.45 SD below the mean, which is the bottom 34% of the sample, and a score of 46 on the OxPos scale would represent 0.26 SD below the mean, which is the bottom 41% of the sample.

The mean score for the 8-item OxPos scale is 17.57 (SD = 7.22) and the mean score of WEMWBS is 42.3 (SD = 11.34), with N = 310. The correlation between the OxPos scale and WEMWBS was moderately high (r = 0.71, p<0). Given the small number of items in the short version, we only used the bottom 25% of the WEMWBS to determine the cut-off point for the short form OxPos scale. ROC analysis identified 15 as an optimal cut-off point (sensitivity = 0.791; specificity = 0.787) with an overall AUC of 0.869. A score of 15 on the OxPos scale would represent approximately 0.36 SD below the mean, which is the bottom 40% of the sample.

*Female between 31 – 50*

The mean score for the 24-item OxPos scale is 54.99 (SD = 22.32) and the mean score of WEMWBS is 43.34 (SD = 12.33), with N = 492. The correlation between the OxPos scale and WEMWBS was moderately high (r = 0.77, p<0). To determine cut-offs for the OxPos scale that will allow us to discriminate participants who have very low or low positive thoughts from those who have moderate or high positive thoughts, we used the bottom 15% and 25% of the WEMWBS (score = 31, score = 36), representing approximately 1 SD and 0.6 SD below the average score in the sample.

ROC analysis identified 41 as an optimal cut-off point (sensitivity = 0.828; specificity = 0.795) with an overall AUC of 0.875 for the bottom 15% of the WEMWBS and 45 as an optimal cut-off point (sensitivity = 0.827; specificity = 0.762) with an overall AUC of 0.869 for the bottom 25% of the WEMWBS. A score of 41 on the OxPos scale would represent 0.63 SD below the mean, which is the bottom 28% of the sample, and a score of 45 on the OxPos scale would represent 0.45 SD below the mean, which is the bottom 34% of the sample.

The mean score for the 8-item OxPos scale is 18.57 (SD = 7.75) and the mean score of WEMWBS is 43.34 (SD = 12.33), with N = 492. The correlation between the OxPos scale and WEMWBS was moderately high (r = 0.76, p<0). Given the small number of items in the short version, we only used the bottom 25% of the WEMWBS to determine the cut-off point for the short form OxPos scale. ROC analysis identified 15 as an optimal cut-off point (sensitivity = 0.846; specificity = 0.713) with an overall AUC of 0.861. A score of 15 on the OxPos scale would represent approximately 0.46 SD below the mean, which is the bottom 35% of the sample.

*Female greater than 51*

The mean score for the 24-item OxPos scale is 60.26 (SD = 20.25) and the mean score of WEMWBS is 47.42 (SD = 11.39), with N = 467. The correlation between the OxPos scale and WEMWBS was moderately high (r = 0.84, p<0). To determine cut-offs for the OxPos scale that will allow us to discriminate participants who have very low or low positive thoughts from those who have moderate or high positive thoughts, we used the bottom 15% and 25% of the WEMWBS (score = 36, score = 40), representing approximately 1 SD and 0.65 SD below the average score in the sample.

ROC analysis identified 48 as an optimal cut-off point (sensitivity = 0.833; specificity = 0.894) with an overall AUC of 0.898 for the bottom 15% of the WEMWBS and 50 as an optimal cut-off point (sensitivity = 0.875; specificity = 0.83) with an overall AUC of 0.912 for the bottom 25% of the WEMWBS. A score of 48 on the OxPos scale would represent 0.61 SD below the mean, which is the bottom 28% of the sample, and a score of 50 on the OxPos scale would represent 0.51 SD below the mean, which is the bottom 31% of the sample.

The mean score for the 8-item OxPos scale is 20.46 (SD = 7.03) and the mean score of WEMWBS is 47.42 (SD = 11.39), with N = 467. The correlation between the OxPos scale and WEMWBS was moderately high (r = 0.83, p<0). Given the small number of items in the short version, we only used the bottom 25% of the WEMWBS to determine the cut-off point for the short form OxPos scale. ROC analysis identified 19 as an optimal cut-off point (sensitivity = 0.82; specificity = 0.877) with an overall AUC of 0.9. A score of 19 on the OxPos scale would represent approximately 0.21 SD below the mean, which is the bottom 39% of the sample.

**Section 9.** Mean (SD) and corresponding percentile score

| Table 9-1. Mean (SD) scores as well as corresponding percentile ranks of the 24-item OxPos measure (subscales and global score) based on complete representative data (N=244, Male, 18 to 30). | | | | | |
| --- | --- | --- | --- | --- | --- |
|  | F1 | F2 | F3 | F4 | Global |
| Mean (SD) | 16.12 (6.86) | 19.24 (7.59) | 12.03 (4.61) | 10.06 (3.73) | 57.45 (20.27) |
| Percentile Rank |  |  |  |  |  |
| 0% | 0.00 | 0.00 | 0.00 | 0.00 | 0.00 |
| 10% | 6.00 | 9.00 | 6.00 | 5.00 | 30.20 |
| 20% | 10.00 | 13.00 | 8.00 | 7.00 | 40.00 |
| 30% | 12.30 | 16.00 | 9.30 | 8.00 | 48.30 |
| 40% | 15.00 | 18.00 | 11.00 | 9.00 | 53.00 |
| 50% | 17.00 | 19.50 | 12.00 | 10.00 | 57.50 |
| 60% | 19.00 | 21.60 | 13.00 | 11.00 | 64.00 |
| 70% | 20.00 | 23.00 | 15.00 | 12.00 | 69.00 |
| 80% | 22.00 | 26.00 | 16.00 | 14.00 | 75.00 |
| 90% | 24.00 | 29.90 | 18.00 | 15.00 | 83.00 |
| 100% | 28.00 | 32.00 | 20.00 | 16.00 | 96.00 |
|  | | | | | |

| Table 9-2. Mean (SD) scores as well as corresponding percentile ranks of the 24-item OxPos measure (subscales and global score) based on complete representative data (N=402, Male, 31 to 50). | | | | | |
| --- | --- | --- | --- | --- | --- |
|  | F1 | F2 | F3 | F4 | Global |
| Mean (SD) | 16.24 (7.11) | 18.87 (8.06) | 11.95 (5.09) | 10.92 (3.76) | 57.97 (21.64) |
| Percentile Rank |  |  |  |  |  |
| 0% | 0.00 | 0.00 | 0.00 | 0.00 | 1.00 |
| 10% | 6.00 | 8.00 | 5.00 | 6.00 | 29.00 |
| 20% | 10.00 | 11.80 | 8.00 | 8.00 | 40.00 |
| 30% | 12.00 | 15.00 | 10.00 | 9.00 | 46.70 |
| 40% | 14.00 | 17.00 | 10.00 | 10.00 | 52.00 |
| 50% | 17.00 | 19.00 | 12.00 | 11.00 | 58.00 |
| 60% | 19.00 | 22.00 | 14.00 | 12.00 | 65.00 |
| 70% | 21.00 | 24.00 | 15.00 | 13.00 | 71.00 |
| 80% | 23.00 | 26.00 | 17.00 | 15.00 | 78.00 |
| 90% | 26.00 | 30.00 | 19.00 | 16.00 | 87.10 |
| 100% | 28.00 | 32.00 | 20.00 | 16.00 | 96.00 |
|  | | | | | |

| Table 9-3. Mean (SD) scores as well as corresponding percentile ranks of the 24-item OxPos measure (subscales and global score) based on complete representative data (N=564, Male, > 50). | | | | | |
| --- | --- | --- | --- | --- | --- |
|  | F1 | F2 | F3 | F4 | Global |
| Mean (SD) | 17.22 (6.68) | 19.98 (7.71) | 13.39 (5.04) | 11.88 (3.31) | 62.47 (20.33) |
| Percentile Rank |  |  |  |  |  |
| 0% | 0.00 | 0.00 | 0.00 | 1.00 | 3.00 |
| 10% | 8.00 | 9.00 | 6.00 | 7.00 | 33.00 |
| 20% | 12.00 | 13.00 | 9.00 | 9.00 | 44.00 |
| 30% | 14.00 | 16.00 | 11.00 | 11.00 | 52.00 |
| 40% | 16.00 | 18.00 | 13.00 | 12.00 | 59.00 |
| 50% | 18.00 | 21.00 | 14.00 | 12.00 | 65.00 |
| 60% | 20.00 | 23.00 | 15.00 | 13.00 | 69.00 |
| 70% | 21.00 | 25.00 | 16.00 | 14.00 | 74.00 |
| 80% | 23.00 | 27.00 | 18.00 | 15.00 | 81.00 |
| 90% | 25.90 | 30.00 | 20.00 | 16.00 | 89.00 |
| 100% | 28.00 | 32.00 | 20.00 | 16.00 | 96.00 |
|  | | | | | |

| Table 9-4. Mean (SD) scores as well as corresponding percentile ranks of the 24-item OxPos measure (subscales and global score) based on complete representative data (N=311, Female, 18 to 30). | | | | | |
| --- | --- | --- | --- | --- | --- |
|  | F1 | F2 | F3 | F4 | Global |
| Mean (SD) | 14.01 (6.94) | 16.13 (7.55) | 11.03 (4.93) | 10.12 (3.89) | 51.29 (20.53) |
| Percentile Rank |  |  |  |  |  |
| 0% | 0.00 | 0.00 | 0.00 | 0.00 | 0.00 |
| 10% | 5.00 | 5.00 | 4.90 | 4.90 | 23.90 |
| 20% | 7.00 | 9.80 | 6.00 | 7.00 | 35.00 |
| 30% | 9.00 | 13.00 | 8.00 | 8.00 | 40.00 |
| 40% | 12.00 | 15.00 | 10.00 | 9.00 | 46.00 |
| 50% | 14.00 | 16.00 | 11.00 | 10.50 | 51.00 |
| 60% | 16.00 | 18.00 | 12.40 | 11.00 | 57.00 |
| 70% | 18.30 | 20.30 | 14.00 | 12.00 | 64.00 |
| 80% | 21.00 | 23.00 | 15.00 | 14.00 | 71.00 |
| 90% | 23.00 | 26.00 | 18.00 | 16.00 | 77.00 |
| 100% | 28.00 | 32.00 | 20.00 | 16.00 | 96.00 |
|  | | | | | |

| Table 9-5. Mean (SD) scores as well as corresponding percentile ranks of the 24-item OxPos measure (subscales and global score) based on complete representative data (N=493, Female, 31 to 50). | | | | | |
| --- | --- | --- | --- | --- | --- |
|  | F1 | F2 | F3 | F4 | Global |
| Mean (SD) | 15.23 (7.29) | 17.62 (8.4) | 10.89 (5.42) | 11.25 (3.88) | 54.99 (22.32) |
| Percentile Rank |  |  |  |  |  |
| 0% | 0.00 | 0.00 | 0.00 | 0.00 | 0.00 |
| 10% | 5.00 | 6.00 | 4.00 | 6.00 | 24.00 |
| 20% | 8.00 | 9.00 | 5.00 | 8.00 | 34.20 |
| 30% | 11.00 | 13.00 | 8.00 | 9.00 | 43.00 |
| 40% | 14.00 | 16.00 | 9.00 | 11.00 | 49.00 |
| 50% | 15.00 | 17.00 | 11.00 | 12.00 | 55.00 |
| 60% | 17.00 | 21.00 | 12.00 | 13.00 | 63.00 |
| 70% | 19.00 | 23.00 | 14.00 | 14.00 | 68.00 |
| 80% | 22.00 | 25.00 | 16.00 | 15.00 | 74.00 |
| 90% | 26.00 | 29.00 | 18.90 | 16.00 | 86.00 |
| 100% | 28.00 | 32.00 | 20.00 | 16.00 | 96.00 |
|  | | | | | |

| Table 9-6. Mean (SD) scores as well as corresponding percentile ranks of the 24-item OxPos measure (subscales and global score) based on complete representative data (N=468, Female, >50). | | | | | |
| --- | --- | --- | --- | --- | --- |
|  | F1 | F2 | F3 | F4 | Global |
| Mean (SD) | 16.68 (6.52) | 19.04 (7.7) | 12.54 (5.13) | 11.99 (3.38) | 60.26 (20.25) |
| Percentile Rank |  |  |  |  |  |
| 0% | 0.00 | 0.00 | 0.00 | 0.00 | 0.00 |
| 10% | 7.00 | 8.00 | 5.00 | 7.60 | 32.00 |
| 20% | 11.00 | 12.00 | 8.00 | 9.00 | 42.00 |
| 30% | 14.00 | 15.00 | 10.00 | 11.00 | 50.00 |
| 40% | 15.00 | 17.00 | 12.00 | 12.00 | 57.40 |
| 50% | 17.00 | 20.00 | 14.00 | 12.00 | 63.00 |
| 60% | 19.00 | 22.00 | 15.00 | 13.00 | 68.00 |
| 70% | 21.00 | 24.00 | 15.00 | 14.00 | 72.00 |
| 80% | 22.00 | 26.00 | 17.00 | 15.00 | 78.00 |
| 90% | 25.00 | 29.00 | 20.00 | 16.00 | 86.00 |
| 100% | 28.00 | 32.00 | 20.00 | 16.00 | 96.00 |
|  | | | | | |

| Table 9-7. Mean (SD) scores as well as corresponding percentile ranks of the 8-item OxPos measure (subscales and global score) based on complete representative data (N=244, Male, 18 to 30). | | | | | |
| --- | --- | --- | --- | --- | --- |
|  | F1 | F2 | F3 | F4 | Global |
| Mean (SD) | 4.76 (2.29) | 4.74 (2.17) | 4.83 (1.99) | 4.98 (2.13) | 19.3 (7.27) |
| Percentile Rank |  |  |  |  |  |
| 0% | 0.00 | 0.00 | 0.00 | 0.00 | 0.00 |
| 10% | 1.00 | 2.00 | 2.00 | 2.00 | 9.00 |
| 20% | 3.00 | 3.00 | 3.00 | 4.00 | 13.00 |
| 30% | 4.00 | 4.00 | 4.00 | 4.00 | 16.00 |
| 40% | 4.00 | 4.00 | 4.00 | 4.00 | 18.00 |
| 50% | 5.00 | 5.00 | 5.00 | 5.00 | 20.00 |
| 60% | 6.00 | 5.60 | 5.00 | 6.00 | 22.00 |
| 70% | 6.00 | 6.00 | 6.00 | 6.00 | 24.00 |
| 80% | 7.00 | 7.00 | 6.00 | 7.00 | 26.00 |
| 90% | 8.00 | 8.00 | 8.00 | 8.00 | 28.00 |
| 100% | 8.00 | 8.00 | 8.00 | 8.00 | 32.00 |
|  | | | | | |

| Table 9-8. Mean (SD) scores as well as corresponding percentile ranks of the 8-item OxPos measure (subscales and global score) based on complete representative data (N=402, Male, 31 to 50). | | | | | |
| --- | --- | --- | --- | --- | --- |
|  | F1 | F2 | F3 | F4 | Global |
| Mean (SD) | 4.64 (2.33) | 4.7 (2.25) | 4.84 (2.13) | 5.34 (2.01) | 19.51 (7.53) |
| Percentile Rank |  |  |  |  |  |
| 0% | 0.00 | 0.00 | 0.00 | 0.00 | 1.00 |
| 10% | 1.00 | 2.00 | 2.00 | 3.00 | 9.00 |
| 20% | 2.00 | 3.00 | 3.00 | 4.00 | 13.00 |
| 30% | 4.00 | 4.00 | 4.00 | 4.00 | 16.00 |
| 40% | 4.00 | 4.00 | 4.00 | 5.00 | 17.00 |
| 50% | 5.00 | 5.00 | 5.00 | 6.00 | 19.00 |
| 60% | 6.00 | 6.00 | 6.00 | 6.00 | 22.00 |
| 70% | 6.00 | 6.00 | 6.00 | 6.30 | 24.00 |
| 80% | 7.00 | 7.00 | 7.00 | 7.00 | 26.00 |
| 90% | 8.00 | 8.00 | 8.00 | 8.00 | 30.00 |
| 100% | 8.00 | 8.00 | 8.00 | 8.00 | 32.00 |
|  | | | | | |

| Table 9-9. Mean (SD) scores as well as corresponding percentile ranks of the 8-item OxPos measure (subscales and global score) based on complete representative data (N=564, Male, >50). | | | | | |
| --- | --- | --- | --- | --- | --- |
|  | F1 | F2 | F3 | F4 | Global |
| Mean (SD) | 5.03 (2.11) | 4.97 (2.13) | 5.4 (2.08) | 5.83 (1.79) | 21.23 (7) |
| Percentile Rank |  |  |  |  |  |
| 0% | 0.00 | 0.00 | 0.00 | 0.00 | 1.00 |
| 10% | 2.00 | 2.00 | 2.00 | 3.00 | 11.00 |
| 20% | 3.00 | 3.00 | 4.00 | 5.00 | 16.00 |
| 30% | 4.00 | 4.00 | 4.00 | 5.00 | 18.00 |
| 40% | 5.00 | 5.00 | 5.00 | 6.00 | 20.00 |
| 50% | 5.50 | 5.00 | 6.00 | 6.00 | 22.00 |
| 60% | 6.00 | 6.00 | 6.00 | 6.00 | 24.00 |
| 70% | 6.00 | 6.00 | 7.00 | 7.00 | 25.00 |
| 80% | 7.00 | 7.00 | 8.00 | 8.00 | 27.00 |
| 90% | 8.00 | 8.00 | 8.00 | 8.00 | 30.90 |
| 100% | 8.00 | 8.00 | 8.00 | 8.00 | 32.00 |
|  | | | | | |

| Table 9-10. Mean (SD) scores as well as corresponding percentile ranks of the 8-item OxPos measure (subscales and global score) based on complete representative data (N=311, Female, 18 to 30). | | | | | |
| --- | --- | --- | --- | --- | --- |
|  | F1 | F2 | F3 | F4 | Global |
| Mean (SD) | 4.03 (2.27) | 4.03 (2.11) | 4.55 (2.09) | 4.97 (2.18) | 17.57 (7.22) |
| Percentile Rank |  |  |  |  |  |
| 0% | 0.00 | 0.00 | 0.00 | 0.00 | 0.00 |
| 10% | 1.00 | 1.00 | 2.00 | 2.00 | 8.00 |
| 20% | 2.00 | 2.00 | 3.00 | 3.00 | 11.00 |
| 30% | 3.00 | 3.00 | 3.00 | 4.00 | 14.00 |
| 40% | 4.00 | 4.00 | 4.00 | 5.00 | 16.00 |
| 50% | 4.00 | 4.00 | 5.00 | 5.00 | 18.00 |
| 60% | 5.00 | 4.00 | 5.00 | 6.00 | 20.00 |
| 70% | 6.00 | 5.00 | 6.00 | 6.00 | 22.00 |
| 80% | 6.00 | 6.00 | 6.00 | 7.00 | 24.00 |
| 90% | 7.00 | 7.00 | 7.10 | 8.00 | 27.00 |
| 100% | 8.00 | 8.00 | 8.00 | 8.00 | 32.00 |
|  | | | | | |

| Table 9-11. Mean (SD) scores as well as corresponding percentile ranks of the 8-item OxPos measure (subscales and global score) based on complete representative data (N=493, Female, 31 to 50). | | | | | |
| --- | --- | --- | --- | --- | --- |
|  | F1 | F2 | F3 | F4 | Global |
| Mean (SD) | 4.34 (2.39) | 4.3 (2.28) | 4.4 (2.29) | 5.54 (2.06) | 18.57 (7.75) |
| Percentile Rank |  |  |  |  |  |
| 0% | 0.00 | 0.00 | 0.00 | 0.00 | 0.00 |
| 10% | 1.00 | 1.00 | 1.00 | 2.10 | 7.10 |
| 20% | 2.00 | 2.00 | 2.00 | 4.00 | 11.00 |
| 30% | 3.00 | 3.00 | 3.00 | 4.00 | 15.00 |
| 40% | 4.00 | 4.00 | 4.00 | 5.00 | 17.00 |
| 50% | 4.00 | 4.00 | 4.00 | 6.00 | 19.00 |
| 60% | 5.00 | 5.00 | 5.00 | 6.00 | 21.00 |
| 70% | 6.00 | 6.00 | 6.00 | 7.00 | 23.00 |
| 80% | 6.80 | 6.00 | 7.00 | 8.00 | 26.00 |
| 90% | 8.00 | 7.00 | 8.00 | 8.00 | 29.00 |
| 100% | 8.00 | 8.00 | 8.00 | 8.00 | 32.00 |
|  | | | | | |

| Table 9-12. Mean (SD) scores as well as corresponding percentile ranks of the 8-item OxPos measure (subscales and global score) based on complete representative data (N=468, Female, >50). | | | | | |
| --- | --- | --- | --- | --- | --- |
|  | F1 | F2 | F3 | F4 | Global |
| Mean (SD) | 4.82 (2.12) | 4.68 (2.07) | 5.08 (2.12) | 5.88 (1.83) | 20.46 (7.03) |
| Percentile Rank |  |  |  |  |  |
| 0% | 0.00 | 0.00 | 0.00 | 0.00 | 0.00 |
| 10% | 2.00 | 2.00 | 2.00 | 3.00 | 10.00 |
| 20% | 3.00 | 3.00 | 3.00 | 4.00 | 14.20 |
| 30% | 4.00 | 4.00 | 4.00 | 5.00 | 17.00 |
| 40% | 4.00 | 4.00 | 5.00 | 6.00 | 20.00 |
| 50% | 5.00 | 5.00 | 5.00 | 6.00 | 22.00 |
| 60% | 6.00 | 6.00 | 6.00 | 6.00 | 23.00 |
| 70% | 6.00 | 6.00 | 6.00 | 7.00 | 24.00 |
| 80% | 7.00 | 6.00 | 7.00 | 8.00 | 26.00 |
| 90% | 8.00 | 8.00 | 8.00 | 8.00 | 29.40 |
| 100% | 8.00 | 8.00 | 8.00 | 8.00 | 32.00 |
|  | | | | | |

References

Aickin, M., & Gensler, H. (1996). Adjusting for multiple testing when reporting research results: the Bonferroni vs Holm methods. *American journal of public health, 86*(5), 726-728. doi:10.2105/ajph.86.5.726

Anderson, D., & Burnham, K. (2004). Model selection and multi-model inference. *Second. NY: Springer-Verlag, 63*(2020), 10.

Bradley, A. P. (1997). The use of the area under the ROC curve in the evaluation of machine learning algorithms. *Pattern recognition*, *30*(7), 1145-1159.

Chen, F. F. (2007). Sensitivity of goodness of fit indexes to lack of measurement invariance. *Structural Equation Modeling: A Multidisciplinary Journal, 14*(3), 464-504. doi:10.1080/10705510701301834

Hanley, J. A., & McNeil, B. J. (1982). The meaning and use of the area under a receiver operating characteristic (ROC) curve. *Radiology*, *143*(1), 29-36.

Holm, S. (1979). A Simple sequentially rejective multiple test procedure. *Scandinavian Journal of Statistics, 6*(2), 65-70. Retrieved from <http://www.jstor.org/stable/4615733>

Jacobucci, R., Grimm, K. J., & McArdle, J. J. (2016). Regularized structural equation modeling. *Structural equation modeling: a multidisciplinary journal*, *23*(4), 555-566.

Metz, C. E. (1978, October). Basic principles of ROC analysis. In *Seminars in nuclear medicine* (Vol. 8, No. 4, pp. 283-298). WB Saunders.

Tibshirani, R. (1996). Regression shrinkage and selection via the lasso. *Journal of the Royal Statistical Society: Series B (Methodological)*, *58*(1), 267-288.

van de Schoot, R., Lugtig, P., & Hox, J. (2012). A checklist for testing measurement invariance. *European Journal of Developmental Psychology, 9*(4), 486-492. doi:10.1080/17405629.2012.686740
